# Broad Influence of Mutant Ataxin-3 on the Proteome of the Adult Brain, Young Neurons, and Axons Reveals Central Molecular Processes and Biomarkers in SCA3/MJD Using Knock-In Mouse Model

**DOI:** 10.3389/fnmol.2021.658339

**Published:** 2021-06-17

**Authors:** Kalina Wiatr, Łukasz Marczak, Jean-Baptiste Pérot, Emmanuel Brouillet, Julien Flament, Maciej Figiel

**Affiliations:** ^1^Institute of Bioorganic Chemistry, Polish Academy of Sciences, Poznań, Poland; ^2^Université Paris-Saclay, Centre National de la Recherche Scientifique, Commissariat à l’Energie Atomique, Direction de la Recherche Fondamentale, Institut de Biologie François Jacob, Molecular Imaging Research Center, Neurodegenerative Diseases Laboratory, Fontenay-aux-Roses, France

**Keywords:** spinocerebellar ataxia type 3 (SCA3), Machado-Joseph disease (MJD), ataxin-3, neurodegenerative, proteome, axon, vesicular transport, energy metabolism

## Abstract

Spinocerebellar ataxia type 3 (SCA3/MJD) is caused by CAG expansion mutation resulting in a long polyQ domain in mutant ataxin-3. The mutant protein is a special type of protease, deubiquitinase, which may indicate its prominent impact on the regulation of cellular proteins levels and activity. Yet, the global model picture of SCA3 disease progression on the protein level, molecular pathways in the brain, and neurons, is largely unknown. Here, we investigated the molecular SCA3 mechanism using an interdisciplinary research paradigm combining behavioral and molecular aspects of SCA3 in the knock-in ki91 model. We used the behavior, brain magnetic resonance imaging (MRI) and brain tissue examination to correlate the disease stages with brain proteomics, precise axonal proteomics, neuronal energy recordings, and labeling of vesicles. We have demonstrated that altered metabolic and mitochondrial proteins in the brain and the lack of weight gain in Ki91 SCA3/MJD mice is reflected by the failure of energy metabolism recorded in neonatal SCA3 cerebellar neurons. We have determined that further, during disease progression, proteins responsible for metabolism, cytoskeletal architecture, vesicular, and axonal transport are disturbed, revealing axons as one of the essential cell compartments in SCA3 pathogenesis. Therefore we focus on SCA3 pathogenesis in axonal and somatodendritic compartments revealing highly increased axonal localization of protein synthesis machinery, including ribosomes, translation factors, and RNA binding proteins, while the level of proteins responsible for cellular transport and mitochondria was decreased. We demonstrate the accumulation of axonal vesicles in neonatal SCA3 cerebellar neurons and increased phosphorylation of SMI-312 positive adult cerebellar axons, which indicate axonal dysfunction in SCA3. In summary, the SCA3 disease mechanism is based on the broad influence of mutant ataxin-3 on the neuronal proteome. Processes central in our SCA3 model include disturbed localization of proteins between axonal and somatodendritic compartment, early neuronal energy deficit, altered neuronal cytoskeletal structure, an overabundance of various components of protein synthesis machinery in axons.

## Introduction

Spinocerebellar ataxia type 3 (SCA3), also known as Machado–Joseph disease (MJD), is a neurodegenerative, late-onset, genetic disorder caused by the expansion of CAG repeats in the coding region of the ATXN3 gene (>50 in patients) (McLoughlin et al., 2020). This mutation leads to an expanded polyglutamine (polyQ) tract in the ataxin-3 protein, causing a toxic gain of function (Orr and Zoghbi, 2007). SCA3 patients most typically display imbalance, motor incoordination, and neurodegeneration in the cerebellum, brainstem, spinal cord, and cerebral cortex (Bettencourt and Lima, 2011). Although the disease-causative mutation is known, the exact molecular and cellular mechanisms of SCA3 remain unclear. Several lines of evidence point to the disruption of axon organization and, therefore, connections between brain structures as one of the main traits in SCA3 pathogenesis (de Rezende et al., 2015; Farrar et al., 2016; Lu et al., 2017). Moreover, axonal impairment was observed in central nervous system (CNS) and peripheries in the form of white mater defects on magnetic resonance imaging (MRI) and peripheral axonal neuropathy in SCA3 patients (D’Abreu et al., 2009; Graves and Guiloff, 2011; Rezende et al., 2018). Axonal impairment may be related to the fact that mutant ataxin-3 aberrantly interacts with microtubules affecting cytoskeleton dynamics in neurons and forms inclusions in axons (Mazzucchelli et al., 2009; Seidel et al., 2010; Chen et al., 2012). Also, mitochondrial impairment is a component of axonal damage in neurodegeneration, and likely plays a role in SCA3 pathogenesis (Da Silva et al., 2019). We recently demonstrated that gross transcriptional changes are absent in the pre-symptomatic SCA3 brain of the Ki91 mouse model; however, dysregulations of proteins and phosphoproteins occurred in these animals, pointing at protein homeostasis as a more general and early trait in disease pathogenesis (Switonski et al., 2015; Wiatr et al., 2019).

The ataxin-3 protein is a particular type of protease, a deubiquitinase, and it has been previously demonstrated that the function in the mutant version of the protein is compromised (Neves-Carvalho et al., 2015), which should have a significant influence on the brain proteome. Therefore, the investigation of proteins seems to be the strongest candidate to discover pathogenic mechanisms or identify the cluster of disease mechanisms. The number of known biomarkers and proteins reliably linked to SCA3 pathogenic processes is still relatively low. Surprisingly, the global model picture of SCA3 disease progression on the protein level, showing the most crucial proteins and pathways in the brain, and neurons, has not been generated and published previously. Therefore in the present work, we aimed to identify protein dysregulations and pathogenic processes in SCA3 on various levels, *in vitro*, and *in vivo*. We designed a research pipeline consisting of three complementary proteomic approaches, mouse behavior, and necessary functional validation assays to unveil with increased resolution the most critical processes influencing the SCA3 pathogenesis. The pipeline combines proteomics paralleled to behavioral milestones of SCA3 Ki91 and correlative proteomics for displaying the proteome linked to disease severity. In the third and the most focused approach, we used targeted proteomics of somatodendritic and axonal compartments from young SCA3 neurons. Our results indicate that the SCA3 disease mechanism is based on the broad influence of mutant ataxin-3 on the neuronal proteome affecting classes of proteins responsible for several central molecular processes. These include disturbed localization of proteins between axonal and somatodendritic compartment, early neuronal energy deficit, altered neuronal cytoskeletal structure, an overabundance of protein synthetic machinery in axons, and altered vesicular trafficking pointing at affected axonal maintenance.

## Results

### The SCA3 in Ki91 Mice Gradually Progresses and Demonstrates Discrete Early and Late Phenotypes

**FIGURE 1:**
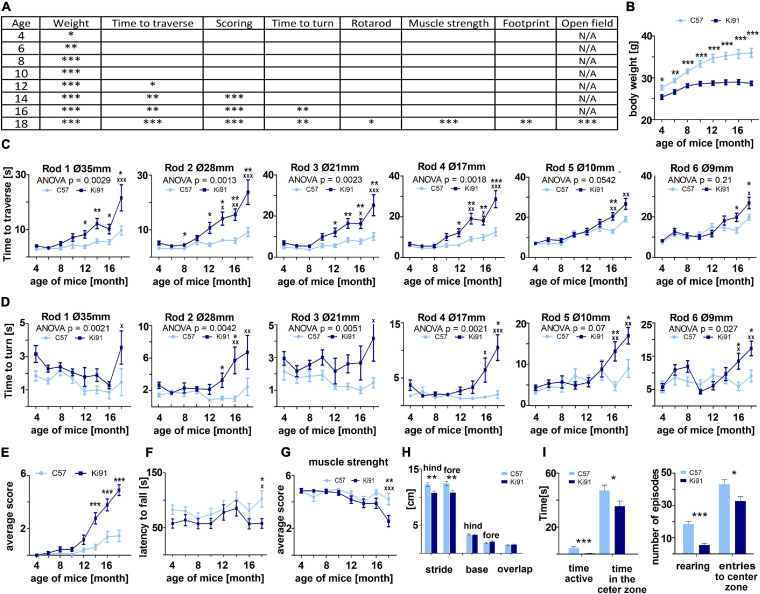
Progressive motor deficits and other disease symptoms in Ki91 SCA3/MJD mice. Ki91 mice presented a gradual decline in several motor and non-motor functions measured by elevated beam walk, rotarod, monitoring of body weight, muscle weakness, and other tests. **(A)** Progressive reduction of body weight gain was observed starting from 4-month-old mice; on average, mut/mut animals at 12 months of age displayed 16.4% less body weight compared to WT animals (*p* < 0.001; two-sample *t*-test) **(B)**. In the elevated beam walk test **(C,D)** “time to turn” and “traverse time” parameters were measured on six rods with decreasing diameter (diameter of rods are indicated by Ø in mm). A total of 12-month-old Ki91 mice needed significantly more time to traverse on rods 1–4, whereas older 16-month-old mice also needed more time on rods 5–6. **(C)** A total of 16-month-old animals needed more time to turn on all rods **(D)**. In the scoring test, 14-month-old Ki91 mice presented symptoms characteristic for SCA3: incoordination, gait disturbances, kyphosis, and hind limb clasping **(E)**. A total of 18-month-old mice showed motor incoordination in accelerated rotarod (4–40 rpm in 9.5 min) **(F)**, muscle weakness **(G)**, and differences in stride length in footprint test **(H)**. A total of 18-month-old mice also presented a cognitive deterioration marked by a decreased amount of time spent in the center zone in the Open field test and a decreased number of rearing **(I)**. Two-way ANOVA with Bonferroni *post hoc* test (*p* ≤ 0.05; total number of biological replicates: *n* = 36, *n* = 18 per genotype), error bars: SEM. Asterisks denotes a two-way ANOVA calculated separately for each test consisted of 4 days at each age (*p < 0.05, **p < 0.01, ***p < 0.001); x-symbol represent two-way ANOVA calculated after completion of testing all ages (^*x*^*p* < 0.05, ^*xx*^*p* < 0.01, ^*xxx*^*p* < 0.001).

We have previously demonstrated that by 2 months of age, Ki91 mice show no motor symptoms or other behavioral abnormalities, and we additionally defined the spectrum of Ki91 motor deficits using 14-month-old animals (Wiatr et al., 2019). Upon completing our longitudinal behavioral tests (4–18 months), we now demonstrate that the homozygous Ki91 model gradually develops disease symptoms resembling disease progression in SCA3 patients. Based on the large volume of longitudinal behavioral data (Figure 1), we have distinguished three stages of behavioral symptom development in Ki91 animals (*n* = 36). The first stage consists of progressive failure to gain body weight (4-month-old) followed by the early symptomatic stage (12-month-old), characterized by gait ataxia and motor incoordination. The final, symptomatic phase (18-month-old animals) is characterized by severe loss of balance and coordination, gait ataxia, dystonia, and muscle weakness (Figure 1A). Reduced rate of body weight gain was observed as the first symptom in 4-month-old Ki91 mice (*p* < 0.05; two-sample *t*-test), and the difference between Ki91 and wild-type (WT) littermates increased with age [*p* < 0.001; two-sample *t*-test, two-way analysis of variance (ANOVA), Bonferroni; Figure 1B]. At the age of 8 months, Ki91 mice stopped gaining weight, and their weight was stable across further ages (Figure 1B) while the WT animals still regularly gained weight. First motor symptoms occurred in 12-month-old animals, which performed worse in the elevated beam walk (Figure 1C). Ki91 took more time to traverse rods (diameter: 35, 28, 21, and 17 mm) and committed more foot slips while performing the task (*p* < 0.05; two-way ANOVA, Bonferroni). As the disease progressed, 14-month-old Ki91 demonstrated overall deterioration of phenotype, including gait disturbances, loss of coordination, and balance, and several Ki91 animals demonstrated hindlimb clasping and kyphosis in the scoring test (*p* < 0.0001; one- and two-way ANOVA, Bonferroni) (Figure 1E). Next, 16-month-old Ki91 mice needed more time to turn on a rod (diameter: 28, 17, 10, and 9 mm) in the elevated beam walk test (*p* < 0.05; two-way ANOVA, Bonferroni), showing loss of balance (Figure 1D). Besides, 18-month-old Ki91 mice demonstrated incoordination in the Rotarod (*p* < 0.05; two-way ANOVA, Bonferroni; Figure 1F), reduced muscle strength (*p* < 0.001; one- and two-way ANOVA, Bonferroni; Figure 1G), shorter stride, which indicated further gait disturbances in the footprint test (*p* < 0.001; two-sample *t*-test, ANOVA, Bonferroni; Figure 1H), and decreased activity and anxiety in the open field test (*p* < 0.05; two-sample *t*-test; Figure 1I). In this last test, Ki91 mice showed significantly less time spent moving (*p* < 0.0001; two-sample *t*-test), as well as decreased vertical activity (rearing) (*p* < 0.0001; two-two-sample *t*-test), a smaller number of the entries, and less time spent in the center zone (*p* < 0.05; two-sample *t*-test). Since mice showed differences in body weight, we ensured that no correlation existed between body weight and behavioral tests in each age to exclude the effect of the animals’ weight on the results (*p* < 0.05, correlation test).

### Ki91 SCA3/MJD Mice Demonstrate Multiple Brain Region Atrophy and the Presence of Atxn3 Inclusions Throughout the Brain

**FIGURE 2:**
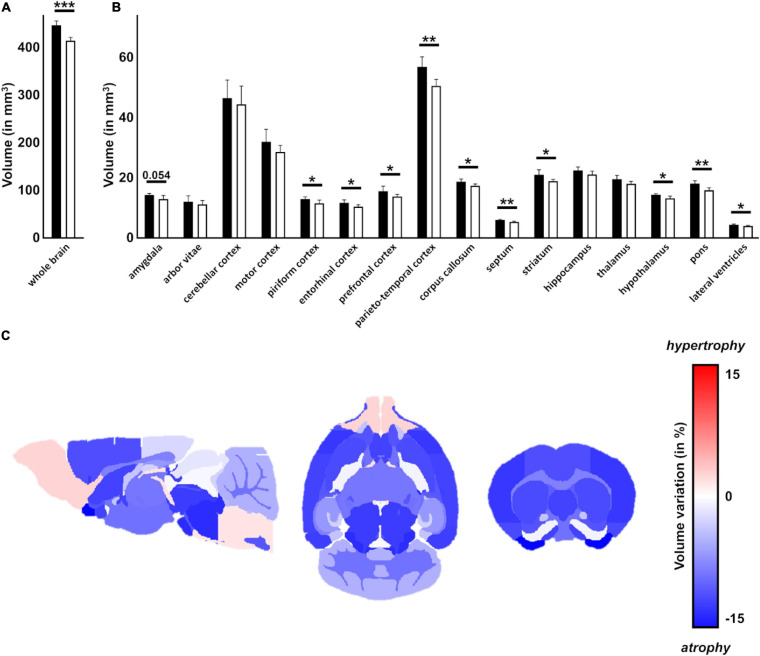
Atrophy of multiple regions including white matter in the Ki91 SCA3/MJD brain. MRI image segmentation was used to measure whole brain volume *ex vivo* of 18-month-old Ki91 and WT mice (*n* = 6 per genotype) and revealed significant global atrophy (–7%, *p* < 0.001) **(A)** and reduction of volume of many regions, such as parieto-temporal cortex (–11%, *p* < 0.01), entorhinal, piriform and motor cortexes (–11% each, *p* < 0.05), corpus callosum (–7,5%, *p* < 0.05), striatum (–11%, *p* < 0.05), septum (–11%, *p* < 0.01), pons (–12%, *p* < 0.01) and hypothalamus (–8%, *p* < 0.05) **(B)**. Schematic representation of brain regions atrophy in Ki91 mice **(C)**. Atrophy of many brain regions might suggest impaired connections between those structures. The gradient of red color is for hypertrophy, the gradient of blue color for the atrophy. Two-sample *t*-test (*p < 0.05, **p < 0.01, ***p < 0.001), error bars: SEM.

Magnetic resonance imaging was used on *ex vivo* brains of 18 months old animals (*n* = 6 per genotype) to measure brain volumes in Ki91 and WT mice (Figures 2A,B). As measured by MRI image segmentation, whole-brain volume showed significant global atrophy of the brain in Ki91 mice (−7%, *p* < 0.001; Figure 2A). When looking at regions volume, several structures seem particularly atrophied as parietal-temporal cortex (−11%, *p* < 0.01), entorhinal, piriform and motor cortexes (−11% each, *p* < 0.05), corpus callosum (−7.5%, *p* < 0.05), striatum (−11%, *p* < 0.05), septum (−11%, *p* < 0.01), pons (−12%, *p* < 0.01), and hypothalamus (−8%, *p* < 0.05) (Figure 2B). There was no significant atrophy in the hippocampus, and cerebellar atrophy was not determined due to MRI coil performance at the end of the brain. Fractional Anisotropy (FA) was also investigated as a biomarker of the integrity of tissue organization. FA was significantly decreased in dentate gyrus (−17%, *p* < 0.01), and stratum granulosum (−19%, *p* < 0.05) (Figure 2C).

**FIGURE 3:**
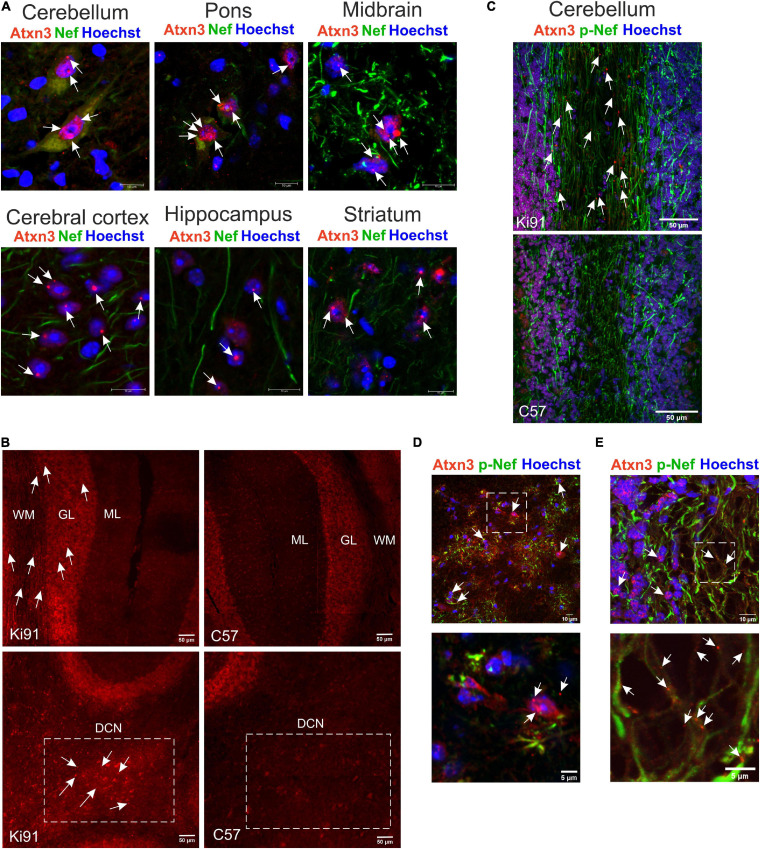
The presence of multi-region intra-nuclear and intra-axonal inclusions in Ki91 SCA3/MJD brain. The ataxin-3 immunostaining of the 18-month-old Ki91 brain sections revealed a large number of cells within the cerebellum, midbrain, pons, cerebral cortex, hippocampus, and striatum with ataxin-3 (red; rabbit anti-ataxin-3 antibody) localizing mainly in the cell nucleus (blue; Hoechst 33342) **(A)**. White arrows indicate large intra-nuclear inclusions; however, many smaller inclusions throughout the brain were present. A rich representation of Atxn3 aggregates (red; rabbit anti-ataxin-3 antibody) was present in DCN, granular layer (gl), and white matter (wm) of the Ki91 cerebellum **(B–D)**. A significant number of small and large ataxin-3 inclusions were detected in the neurites (green; Smi-32, pan-neuronal or Smi-312, axon-specific antibody) of the Ki91 cerebellum **(C,E)**. White box and arrows in **(D)** mark prominent Atxn3 aggregates in the DCN area (lower panel represents the magnification of selected region in the upper panel). White box and arrows in **(E)** mark the magnified area containing multiple intra-axonal inclusions of Atxn3 (lower panel). Scale bars: 50 μm **(B,C)**; 10 μm **(A)**; 5 μm on inserts **(D,E)**. *N* = 4 biological replicates; at least 4 pictures per brain region of each kind were collected.

Next, we have performed brain sections from 18-month-old Ki91 and staining using the anti-ataxin-3 antibody (Figure 3). We found that all brain areas, including the cerebral cortex, striatum, midbrain, hippocampus, cerebellum, DCN, and pons, demonstrated intensive positive signals in the form of both inclusions and diffuse staining of cellular structures (*n* = 4; Figures 3A–E). In particular, intense anti-ataxin-3 staining was present in cell nuclei, and many cells demonstrated large inclusions (Figure 3A). Interestingly, a significant number of smaller and larger ataxin-3-positive inclusions were scattered along the neurites (particularly axons labeled by SMI-312 axonal marker) in the cerebellum (Figures 3C,E). Although we could not measure cerebellum atrophy by MRI, we determined that the inclusions are rich in the cerebellum, indicating an intense pathogenic process. In the cerebellum, the large inclusions were predominantly present in the DCN area, white matter, and in some cells located in the granular layer (Figures 3B,D). Many of the atxn3 inclusions were ubiquitinated (Supplementary Figures 1A,B, 2). Considering widespread brain atrophy and the inclusions in all regions, we reasoned that the brain pathogenic SCA3 processes are multi-region and multi-thread. Therefore we selected the cerebellum and cerebral cortex to represent both classically reported SCA3 pathogenesis in the hindbrain and postulated SCA3 pathogenesis in more frontal parts of the brain.

### Proteomics Parallel to Behavioral Milestones Implicates Disturbed Metabolism, Cytoskeleton, and Vesicular Trafficking in Ki91 SCA3/MJD Mice

Parallel to behavioral experiments and based on the occurrence of symptoms in behavioral tests, we have selected four mouse ages (4, 10, 12, and 14-month-old, *n* = 4 per genotype) to analyze proteomic changes in the cerebral cortex (entire region collected for analysis) and cerebellum. In detail, 4-month-old Ki91 animals displayed reduced body weight gain; a 10-months stage was just before the onset of motor symptoms, while 12 and 14-month-old mice showed a decline in motor functions (Figure 1); therefore, the protein dysregulation in those ages would be most relevant. The principal component analysis (PCA) mostly demonstrated distinct clustering of the Ki91 and WT datasets (Supplementary Figures 3A,B). Altogether, we identified 115, 126, 212, and 75 proteins that were significantly dysregulated (*p* < 0.05; two-sample *t*-test) in the cerebellum of 4-, 10-, 12-, and 14-month-old Ki91 mice, respectively (Supplementary Table 1 and Supplementary Figure 3C). In comparison, 178, 89, 170, and 279 proteins were significantly dysregulated (*p* < 0.05; two-sample *t*-test) in the cerebral cortex of 4-, 10-, 12-, and 14-month-old Ki91 animals, respectively (Supplementary Table 2 and Supplementary Figure 3C).

**FIGURE 4:**
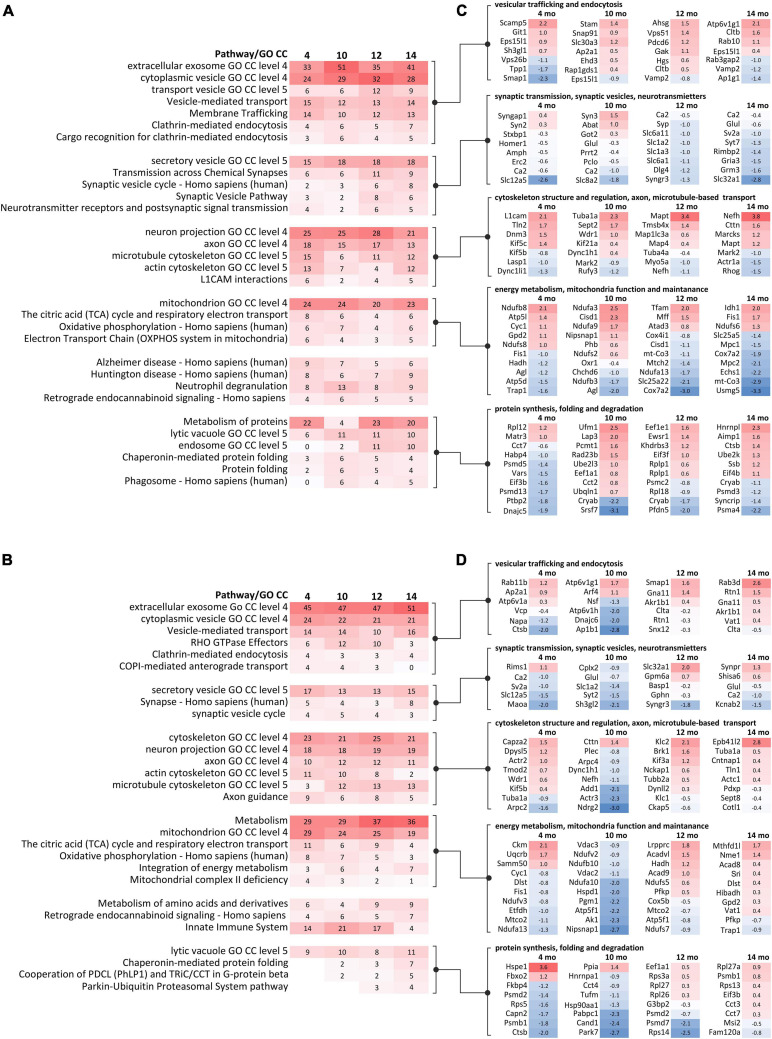
GO term and pathway analysis of dysregulated proteins identified in proteomics at behavioral milestones in the Ki91 SCA3/MJD mouse brain implicate vesicular transport, cytoskeleton, protein metabolism, and mitochondrial function. Bioinformatic analysis of biological pathways and subcellular localization of dysregulated proteins (*p* < 0.05; two-sample *t*-test, *n* = 4 per genotype) during disease progression in the cerebral cortex **(A)** and cerebellum **(B)** was assessed using ConsensusPath database (CPDB; pathways *p*-value cut-off < 0.01; GO terms cellular component, *p*-value cut-off < 0.01, level 3, 4). The heatmaps present changes of pathways and subcellular localization involving dysregulated proteins (*p* < 0.05; two-sample *t*-test) over the course of the disease in four tested ages (4, 10, 12, and 14-month-old) by showing what percentage of dysregulated proteins in each set is assigned to the particular pathway or subcellular region (numbers). The gradient of red color denotes a higher percentage of dysregulated proteins (more intense color) to a lower percentage of dysregulated proteins (less intense color). The five groups of pathways and GO terms arranged in blocks in **(A,B)** are linked to the corresponding lists of dysregulated proteins (*p* < 0.05; two-sample *t*-test) with the highest log2-FC in each tested age, assigned to five arbitrary categories in the cerebral cortex **(C)** and cerebellum **(D)**. The categories were formed based on the analysis of pathways and GO terms and are as follows: (1) vesicular trafficking and endocytosis; (2) synaptic transmission, synaptic vesicles, and neurotransmitters; (3) cytoskeleton structure and regulation, axon, and microtubule-based transport; (4) energy metabolism, mitochondria function, and maintenance; and (5) protein synthesis, folding, and degradation. The lists of dysregulated proteins are also presented as heatmaps with numbers representing log2-FC **(C,D)**. The gradient of red color is for upregulated proteins, blue color for downregulated proteins.

In order to reveal molecular pathways and cellular compartments (CCs), which might contribute to the SCA3 pathogenesis, we performed an analysis of dysregulated proteins using the ConsensusPath database (CPDB; pathways and GO CC for each age; level 4 and 5; *q* < 0.01). For simplicity, we have arbitrarily grouped pathways and CCs identified in CPDB into five categories for the cerebral cortex (Figure 4A) and cerebellum (Figure 4B) based on their biological relations and similarity. Lists of proteins with the highest log2-fold changes (log2-FC) belonging to five categories are presented for the cerebral cortex (Figure 4C) and cerebellum (Figure 4D).

The first category was related to vesicular transport and endocytosis. Cytoplasmic vesicles and extracellular exosomes were the localization for 24–32 and 33–51% of dysregulated proteins in every dataset from the cortex (Figure 4A) and similarly 21–24 and 45–51% in the cerebellum (Figure 4B). Moreover, vesicle-mediated transport and clathrin-mediated endocytosis are predicted to be disturbed in both the cerebral cortex (Vamp2, Cltb, and Rab10; Figure 4C) and cerebellum (Rab11b, Napa, Nsf, and Rab3d; Figure 4D).

The second category included dysregulated proteins involved in synaptic transmission. There was a relative increase in the number of altered proteins in older 12–14 month-old Ki91 mice in the cerebral cortex compared to younger ages (4–10-month-old). For instance, pathways such as “transmission across chemical synapses” contained ∼ 6% of dysregulated proteins in 4–10-month-old vs. ∼ 10% in 12–14% and “synaptic vesicle cycle” ∼ 3% vs. 7%, respectively (Figure 4A). Proteins related to SNARE complex and synaptic vesicles such as Syn3, Syp, Sv2a, and Syt7 were dysregulated (Figure 4C). Interestingly, most of the proteins with a function in the synapse showed decreased levels in the cerebral cortex of older 12–14-month-old Ki91 mice (Figure 4C). In the cerebellum, dysregulated proteins of synaptic vesicles or synapses were less frequent compared to the cortex (Figure 4B) and included Sv2a, Syt2, and Kcnab2 (Figure 4D). In both tested tissues, downregulation of Car2, Qdpr, and Glul, were detected in most of the tested ages (Figures 4C,D), which we also validated using vacuum dot-blot assay (*p* < 0.05; two-sample *t*-test; Supplementary Figure 4). Glul, Car2, and Qdpr play a role in the metabolism of amino acids and neurotransmitters, and Qdpr level is slightly and gradually dropping down across ages (Supplementary Tables 1, 2).

The third category was referring to altered proteins playing a role in regulating cytoskeletal structure and function. Dysregulated proteins localized to neuronal projections (axons and dendrites) constituted between 21 and 28% in the cortex and ∼ 18% in the cerebellum (Figures 4A,B). Proteins assigned to cytoskeletal localization included actin (cortex: Actr1a; cerebellum: Arpc2 and Actr3), microtubules (cortex: Tuba1a and Mapt) and neurofilaments (cortex and cerebellum: Nefh) (Figures 4C,D). Disturbances of the cytoskeleton structure may lead to cellular transport defects, and there were many proteins directly regulating anterograde and retrograde axonal transport. For instance, in the cerebral cortex, dysregulated proteins involved in anterograde microtubule-based transport were Kif5c, Kif5b, and Kif21a and retrograde transport: Dnm3, Dync1li1, Dync1h1, and microtubule-associated proteins: Map1lc3a and Mark2 (Figure 4C). In the cerebellum, proteins involved in the anterograde microtubule-based transport included Kif5b, Klc1, Klc2, and Kif3a, and in retrograde transport Dync1h1 and Dynll2 (Figure 4D).

The fourth category involved many dysregulated proteins related to energy metabolism and mitochondria maintenance (Figures 4A–D). Proteins predicted to localize in mitochondrion were between 20–24 and 19–29% in the cortex and cerebellum, respectively (Figures 4A,B). The top pathway involving dysregulated proteins in the Ki91 cerebellum was metabolism (Figure 4B). Moreover, the number of proteins dysregulated in the cerebellum and associated with metabolism increases with age (4–10-month-old: ∼ 29%; 12–14-month-old: ∼ 36%; Figure 4B). Proteins related to tricarboxylic acid cycle (TCA) and oxidative phosphorylation (OXPHOS) are dysregulated in the cerebral cortex (mitochondrial proteins Ndufb8 with; Ndufa3, Ndufs6, and mt-Co3; Figure 4C) and cerebellum (Ndufa13, Ndufa10, and Ndufs7; Figure 4D). There were also dysregulated proteins responsible for mitochondria fission (Fis1 and Mff) and mitochondrial chaperons (Hspd1 and Trap1) (Figures 4C,D). Also, several proteins with altered levels mostly related to metabolism were linked to Alzheimer’s and Huntington disease in both the cerebellum and cerebral cortex (Figures 4A,B).

The fifth category included dysregulated proteins involved in protein metabolism. In the case of the cerebral cortex, there are 4–23% dysregulated proteins involved in the metabolism of proteins (Figure 4A). Noteworthy, the number of dysregulated proteins predicted to localize in lysosomes, endosomes, and phagosomes is higher in the cortex of older mouse (Figures 4A,B). Similarly, the number of altered proteins involved in protein folding and “Parkin-Ubiquitin Proteasomal System pathway” is increasing with age in the Ki91 cerebellum (Figure 4B). Dysregulated proteins in this category involve also various ribosomal subunits and translation factors (cortex: Rpl12 and Eif3b; cerebellum: Rpl27a), proteasome subunits (cortex: Psmd13 and Psma4; cerebellum: Psmb1 and Psmd7), and chaperons (cortex: Dnajc5 and Cryab; cerebellum: Hspe1 and Park7) (Figures 4C,D). Downregulation of Cryab, which was one of the most frequently identified proteins implicated in the pathology of neurodegenerative disease (Zhu and Reiser, 2018), was confirmed with vacuum dot blot assay (*p* < 0.05; two-sample *t*-test) in the cerebral cortex (10, 14, and 18-month-old; Supplementary Figure 4A) and cerebellum (18-month-old; Supplementary Figure 4B). Of note, most of the proteasome subunits and chaperons demonstrated decreased levels in both tested tissues (Figures 4C,D).

### Proteomics Correlative With SCA3 Behavioral Phenotype in 18-Month-Old Ki91 Mice Reveals Dysregulated Proteins Involved in Transport, Cytoskeleton, Synapse, and Mitochondria

Our correlative proteomic approach aimed to discover dysregulated proteins in the brain of animals that demonstrated consistent behavioral phenotype to establish a more exact relationship between the protein biomarkers and the degree of SCA3 disease severity (Supplementary Figure 5). Therefore, after the endpoint of behavioral studies (18 months), the animals were grouped by the intensity of phenotype, and three phenotype subgroups were established in the Ki91 cohort: severe, moderate, and mild (*n* = 4). The selection was based on score assessment on the 0–5 scale (Kruskal–Wallis test, *p* < 0.05) (Supplementary Figure 6A). The scores 0 or 1 represented the “mild phenotype” group, score 2 or 3 was the “moderate phenotype” group, and 4 or 5 was the “severe phenotype” group. The cerebellum and cerebral cortex were collected from each group, and the tissues were subjected to proteomics (Supplementary Table 3 and Supplementary Figures 5A,B). The analysis of dysregulated proteins revealed that protein levels in the moderate group were mostly in between the level of the severe and mild group, demonstrating a gradual pattern of protein dysregulation in Ki91 mice (Supplementary Figures 6B,C). Therefore in further data processing, we focused on the protein levels in severe and mild phenotype.

We found that in the cerebellum of the Ki91 animals with the severe phenotype (*n* = 4), there were heat shock protein Dnaja1 and a nuclear importin Kpna4 upregulated compared to WT animals (WT; *n* = 10) (Supplementary Figure 6B). Another member of the importin family, Kpna3, was previously shown to control the nuclear localization of ataxin-3 (Sowa et al., 2018). There were also five upregulated proteins in the mild phenotype (*n* = 4) – Pvalb, Pak1, Kif5c, and neurofilaments (Nefl and Nefm). Pak1, a kinase implicated in vesicle-mediated transport and regulation of cytoskeleton dynamics, and Kif5c involved in microtubule-based transport, were also downregulated in severe phenotype (Supplementary Figure 6B). Moreover, in the severe group, there were also other downregulated proteins playing a pivotal role in transport along the microtubule (Klc1, Kif5c, Vatl1, and Map4) and notably Purkinje cell protein 4 (Pcp4), which was not dysregulated in mild phenotype (Supplementary Figure 6B). Noteworthy, mitochondrial proteins were downregulated in both severe and mild phenotypes. Mitochondrial chaperone Timm9 showed lower levels in the “severe” group, while Ndufb5 and Fasn were downregulated in the “mild” group (Supplementary Figure 6B).

In the cerebellar cortex, the only upregulated protein was a subunit of mitochondrial ATP synthase, Atp5a1 (Supplementary Figure 6C). Proteins commonly downregulated in both severe and mild phenotype (Supplementary Figure 6C) included Glul, already identified in the proteomics performed parallel to behavioral milestones and myelin protein Cnp, ubiquitinating enzyme Ube2n, transcription factor Tceb1, and Snx12 involved in intracellular trafficking. In the mild group only, there were downregulated proteins associated with synaptic vesicles and post-synaptic density (Akap5, Bsn, Pclo, and Gprin1; Supplementary Figure 6C). Whereas in a “severe” group, there were downregulated proteins related to intracellular transport and cytoskeleton (Snx12, Dlg3, Dnm2, Cap2, and Atcay), and mitochondrial protein Cox7a2 (Supplementary Figure 6C).

Altogether, we identified a set of proteins involved in intracellular and microtubule-based transport downregulated in the cerebellum and cerebral cortex of Ki91 mice displaying severe phenotype. Downregulated proteins in the “mild” phenotype were related to mitochondria in the cerebellum and synapse in the cerebral cortex. Proteins upregulated in the cerebellum were associated with cytoskeleton and neurofilaments in the “mild” group. Cellular processes and compartments associated with dysregulated proteins in “severe” phenotype were related to mitochondria, intracellular and microtubule-based transport, synaptic vesicles, and the cytoskeleton. Most of these processes and compartments were also identified in our approach parallel to behavioral milestones. An essential neuronal structure, which connected those terms, is the axon, which requires high energy production, highly efficient trafficking of cargoes toward its end, and is a location of synaptic vesicle release. Therefore, we further decided to go to the next level of neuronal complexity in our proteomic investigation and selectively explore proteins dysregulated in the SCA3 axons.

## Targeted Axonal Proteomics Indicates the Abnormal Localization of Proteins Between Axons and Soma Such as Cytoskeletal, Vesicular Proteins, and Highly Increased Translation Machinery in Ki91 SCA3 Neurons

**FIGURE 5:**
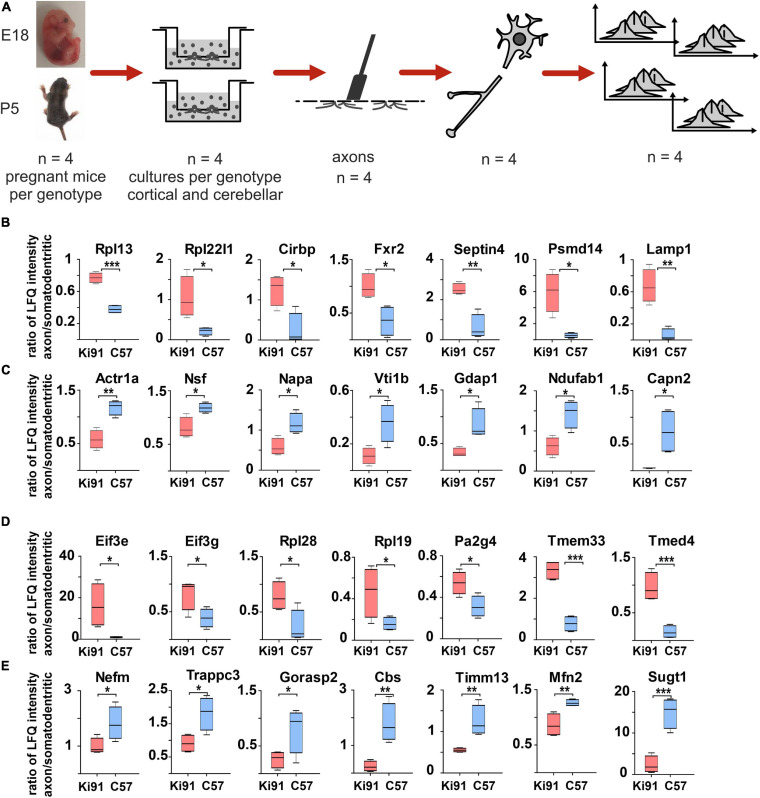
Targeted axonal proteomics reveals sets of proteins enriched or depleted in the Ki91 SCA3/MJD cerebellar and cortical axons. Identification of proteins differentially localized in SCA3 neuronal compartments was performed by isolating two neuronal fractions (axonal and somatodendritic) from the same culture (Boyden chambers) obtained from E18 embryo (cortical) or P5 pups (cerebellar) – derived neurons. In brief, neurons were seeded on the inner side of the Boyden chamber inserts with a 1-μm porous filter. After the period of neuronal differentiation and axonal growth through and toward the outer side of the insert membrane, both the inner side of the insert (somatodendritic) and the outer side (axonal) were scraped, and the fractions were collected for MS/MS analysis **(A)**. Graphs present proteins with abnormal localization between axon and soma with the highest ratio between those compartments (*p* < 0.05; two-sample *t*-test) in the primary cerebellar **(B,C)** and cortical Ki91 neurons **(D,E)**. In the P5-derived Ki91 cerebellar axons, there were enriched proteins related to ribosomes (Rpl13 and Rpl22l1), RNA-binding (Cirbp and Fxr2), and proteasome or lysosome (Psmd14 and Lamp1) **(B)**. Proteins depleted in the Ki91 cerebellar axons involved important modulators of vesicular transport (Actr1a, Nsf, Napa, and Vti1b) **(C)**. In the E18-derived Ki91 cortical axons, there were enriched translation factors (Eif3e and Eif3g), ribosome subunits (Rpl28 and Rpl19), RNA-binding (Pa2g4), and ER and Golgi (Tmed4 and Tmem33) **(D)**. Proteins that were depleted in the Ki91 cortical axons included neurofilament (Nefm), related to intracellular trafficking (Trappc3 and Gorasp2), and mitochondria (Timm13 and Mfn2) **(E)**. two-sample *t*-test (*p < 0.05, **p < 0.01, ***p < 0.001), *n* = 36, *n* = 4 per phenotype, error bars: SEM.

Our next aim was to define with high precision the specific processes which highly contribute to axonal dysfunction in SCA3 already at a very early level of neuronal maturation stage, where no neuronal damage takes place, and therefore no secondary post-symptomatic events occur. Therefore, we performed the analysis of enriched or decreased proteins in axons of the cerebellar (young postnatal; P5) and cortical neurons (embryonal; E18) growing in Boyden chambers (*n* = 4 per genotype; Figure 5A).

Both somatodendritic and axonal protein extracts were subjected to MS/MS proteomics, and the “raw levels” for each protein for both compartments were determined (Supplementary Table 4). To normalize the protein levels within fractions originating from neurons on the same filter, we calculated the protein level ratio between the axonal and somatodendritic compartments for each WT and Ki91 sample/filter (Supplementary Table 4). Such normalization has proved very solid since the comparison of soma/axon ratios within the single genotype (WT or Ki91) demonstrated that the most significant difference was the abundance of nuclear proteins, such as histones, and nuclear enzymes characteristic for the somatodendritic compartment (ratio axon to soma < −3; Supplementary Table 4). Coherently, the PCA graph demonstrates a clear separation between axonal and somatodendritic samples (Supplementary Figures 7C,D).

**FIGURE 6:**
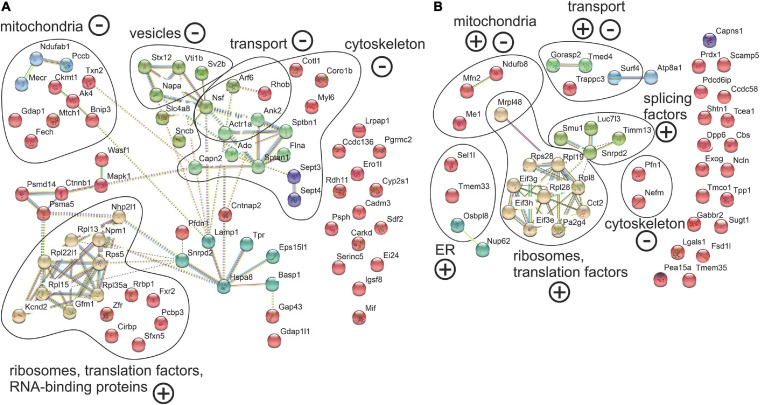
Abnormal localization between axon and soma in the cerebellar and cortical neurons of Ki91 SCA3/MJD build an intensely interconnected network of proteins of translation machinery, vesicles, and transport machinery, cytoskeletal and mitochondrial proteins. The network of proteins displaying altered ratio between axon and soma in cerebellar **(A)** and cortical neurons **(B)** of Ki91 mice were generated using String database and clustering (https://string-db.org/). Number of proteins enriched in cerebellar axons *n* = 34 (ratio axon/soma > 1, *p* < 0.05; two-sample *t*-test); reduced in cerebellar axons *n* = 41 (ratio axon/soma < 1, *p* < 0.05; two-sample *t*-test); enriched in cortical axons *n* = 27 (ratio axon/soma > 1, *p* < 0.05; two-sample *t*-test); reduced in cortical axons *n* = 20 (ratio axon/soma < 1, *p* < 0.05; two-sample *t*-test), four biological replicates per genotype. Distinct colors denote clusters of proteins generated by the String algorithm, which grouped functionally associated proteins into specific sets, such as translational machinery (ribosomes and translation factors), cytoskeletal-modulators, and vesicular proteins. Functional groups are marked and named based on GO annotations (BP, M Level 3, 4, and 5; *q* < 0.01) using CPDB or GO term search if not available in CPDB. “+” and “–” denote for enrichment or depletion of protein groups in axons based on F.C. of the majority of proteins in the group. Blue and pink edges represent known interactions, green, red, and dark blue represent predicted interactions, while black and purple are other interactions.

Subsequently, we compared protein level ratios between WT and Ki91 SCA3/MJD neurons to calculate the fold change and estimate if the protein was enriched or depleted in axons (*p* < 0.05; two-sample *t*-test; Supplementary Table 4). In the cerebellar neurons, there were 34 proteins enriched (ratio axon/soma > 1, *p* < 0.05; two-sample *t*-test; *n* = 4) and 41 proteins depleted (ratio axon/soma < 1, *p* < 0.05; two-sample *t*-test; *n* = 4) in Ki91 vs. WT axons (Figures 5B,C and Supplementary Tables 5, 6). There were 27 and 20 such proteins in cortical neurons, respectively (Figures 5D,E and Supplementary Tables 7, 8). The analysis of proteins in both cerebellar and cortical Ki91 axons revealed a common functional characteristic. The majority of the axon-enriched proteins in SCA3 samples were related to the upregulation of protein production and degradation (Figures 5B,D, 6A,B and Supplementary Tables 6, 8). Strongly upregulated was the translation machinery, in particular the initiation step and nonsense-mediated decay together with the RNA binding proteins, including Cirbp (FC = 4.92; Figure 5B), which stabilizes mRNAs involved in cell survival during stress (Liao et al., 2017). Highly upregulated were the proteasome subunits and chaperones enriched in cerebellar Ki91 axons (Psmd14, Psma5, and Hspa8; Supplementary Table 6) and cortical Ki91 axons (Sel1l and Cct2; Supplementary Table 8), which altogether might be related to the neuronal response to cellular stress and misfolded proteins in SCA.

In contrast, proteins that were reduced in Ki91 axons comparing to WT were in the majority related to the actin cytoskeleton and vesicle-mediated transport, which may indicate the axonal structural dysfunction (Figures 5C,E, 6A,B and Supplementary Tables 5, 7). In Ki91 cerebellar axons, there were reduced levels of 13 proteins related to cytoskeleton, including 8 proteins regulating actin function (Figures 5C, 6A and Supplementary Table 5). The actin cytoskeleton is involved not only in maintaining the proper structure of the axon but also plays a pivotal role in the short-range transport, selection of cargoes targeted to the axon tip, formation of synapses, and scaffold for the mitochondria in the growth cone (Kevenaar and Hoogenraad, 2015). Along this line, we identified eight mitochondrial proteins with a lower ratio axon vs. soma in cerebellar SCA3 samples as compared to WT (Figures 5C, 6A and Supplementary Table 5). Altogether, this may indicate the disrupted delivery of mitochondria to the proper axon area due to the deregulated function of the actin cytoskeleton. Moreover, there are also proteins with a reduced level in cerebellar Ki91 axons directly related to intracellular trafficking (Actr1a, Ank2, Arf6, Nsf, and Rhob), synaptic/SNARE vesicles (Sv2b, Napa, and Vti1b), and COPI-mediated transport from ER to Golgi (Napa, Actr1a, Nsf, Sptan1, Sptbn, and Ank2) (Figures 5C, 6A and Supplementary Table 5).

In cortical Ki91 axons, there were generally fewer proteins identified with lower ratio axons to the soma (Figure 5E and Supplementary Table 7). However, there were also proteins related to mitochondria, transport, and cytoskeleton, including neurofilament medium-chain (Nefm), playing an essential role in axonal function (Figures 5E, 6B and Supplementary Table 7).

### Impairment of Mitochondrial Metabolic Potential in Ki91 SCA3/MJD Primary Cerebellar Neurons

**FIGURE 7:**
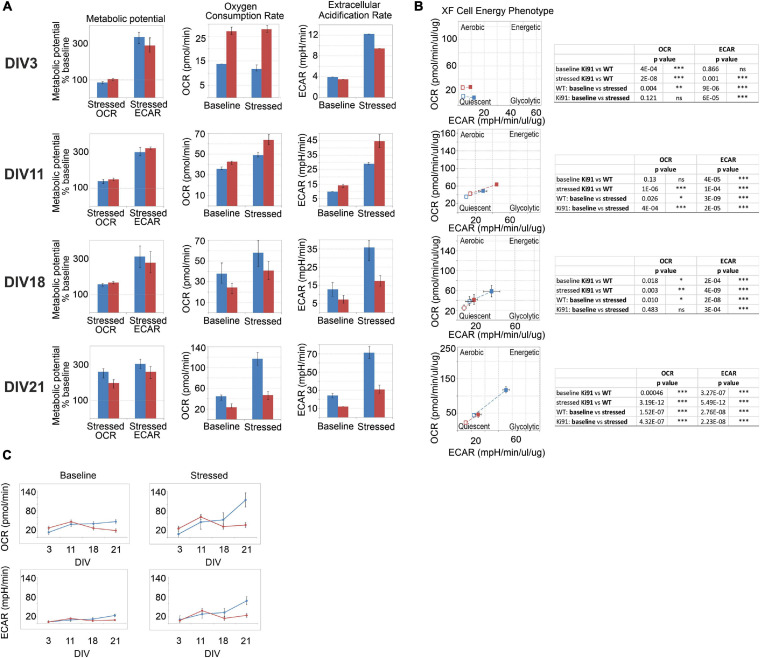
The energy metabolism measured by Seahorse XFp Cell Energy Phenotype Test is impaired in the P5 cerebellar neurons of Ki91 SCA3/MJD. The assays were performed with cerebellar neurons at DIV3, 11, 18, and 21. The rate of mitochondrial respiration (OCR, oxygen consumption rate) and glycolysis (ECAR, extracellular acidification rate) were measured under baseline and stressed conditions, which were evoked by specific stressors: 1 μM of oligomycin and 1 μM of FCCP. The bioenergetic parameters are shown for OCR (pmol/min), ECAR (mpH/min), and metabolic potential [(stressed OCR or ECAR/baseline OCR or ECAR) × 100%)] in **(A)**. In DIV3 and DIV11, OCR in neurons is elevated in Ki91 compared to WT (*p* < 0.001; two-sample *t*-test), but in later stages (DIV21), both OCR and ECAR are severely decreased in Ki91 neurons (*p* < 0.001; two-sample *t*-test) under both baseline and stressed conditions **(A)**. WT cerebellar neurons transform from the quiescent phenotype in DIV3 toward energetic in DIV21, but Ki91 does not undergo such transformation **(B)**. The profiles of OCR and ECAR during tested developmental stages *in vitro* are presented in **(C)**. The blue color is for WT neurons; red is for Ki91 neurons. Experiments were performed in *n* = 6 per genotype and stage. Two-sample *t*-test (*p < 0.05, **p < 0.01, ***p < 0.001), error bars: SEM.

Spinocerebellar ataxia type 3 patients show decreased BMI, and similarly, SCA3 Ki91 mice fail to gain weight. Moreover, our proteomic approaches identified mitochondria and energy metabolism failure in Ki91. The direct investigation of energy metabolism in presymptomatic young SCA3 neurons was not performed previously; therefore, it is unknown if the mitochondrial phenotype occurs early in SCA3 and if mitochondrial dysfunction is a primary contributor to the SCA3 pathology or only the secondary result of putative mice and patient dysphagia. To address whether energy metabolism is disturbed in Ki91 neurons, we performed Cell Energy Phenotype Test with Seahorse XFp analyzer. The assay was performed on primary cerebellar neurons in days *in vitro* 3 (DIV3), 11, 18, and 21 (*n* = 6 per genotype and stage; Figures 7A–C). The oxygen consumption rate (OCR) and extracellular acidification rate (ECAR) were measured over time at baseline and stressed conditions (injection of oligomycin and FCCP mixture) (Supplementary Figure 8). Then, the metabolic potential of neurons was evaluated by calculating changes after stress conditions relative to baseline. The test demonstrated a significant increase in the ECAR metabolic potential in both Ki91 and WT neurons in every tested stage (*p* < 0.0001; two-sample *t*-test; Figures 7A,B). Likewise, an increase of the OCR metabolic potential was observed in WT neurons and Ki91 neurons, but only in DIV11 and 21 (*p* < 0.05; two-sample *t*-test; Figures 7A,B). Furthermore, under stressed conditions, the levels of ECAR were diminished in Ki91 neurons compared to WT in DIV3, 18, and 21 (*p* < 0.001; two-sample *t*-test; Figures 7A,C). Interestingly, both baseline and stressed OCR was increased in Ki91 neurons compared to WT in DIV 3 and 11 (*p* < 0.0001; two-sample *t*-test; DIV11 baseline *p* = 0.13; Figures 7A,C), while in later stages, the trend was reversed and in DIV18 and 21 it was significantly decreased (*p* < 0.01; two-sample *t*-test; Figures 7A,C).

Overall, these data demonstrate that the potential for energy production is impaired in cerebellar Ki91 neurons. The glycolysis rate shows fluctuations in both baseline and stressed conditions. On the other hand, mitochondrial respiration shows a coherent pattern, which starts with an increase under both tested conditions, following by a decrease. The study demonstrates that energy deficit occurs early in SCA3 neurons, and therefore it is not a secondary event resulting from previous neuronal damage.

### Assessment of Vesicle State in Neurites of Ki91 SCA3/MJD Cerebellar Neurons

**FIGURE 8:**
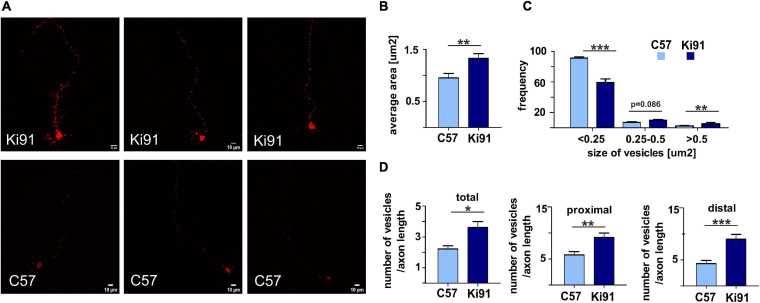
Accumulation and enlargement of vesicles in Ki91 SCA3/MJD P5 cerebellar neurons. To investigate if the consensus vesicular and transport process identified by various types of proteomics in Ki91 impacts vesicle appearance and number, we transfected the primary cerebellar neurons (DIV8-9) with RFP-labeled Rab7. The Rab7 was selected as a useful vesicle tag since it is often recruited to several types of vesicles, including late endosomes and lysosomes (red). Representative images show fluorescently tagged vesicles in cerebellar neurons from Ki91 mice (upper panel) and WT neurons (lower panel) **(A)**. The sizes and the number of tagged vesicles were quantified using ImageJ. The average area of fluorescently tagged vesicles was 1.33 μm^2^ (*n* = 45) for Ki91, 0.95 μm^2^ (*n* = 34) for WT (*p* < 0.01; two-sample *t*-test) **(B)**. The size distribution of tagged vesicles in Ki91 cerebellar neurons showed a shift from smaller to larger sizes in comparison to WT (*p* < 0.001; two-sample *t*-test) **(C)**. The number of vesicles per axon length was higher in Ki91 neurons, also measured separately for both proximal and distal parts (defined as 1/8 of the axon length starting either from the axon hillock or axon tip) **(D)**. The measurements were from five independent experiments, with 5–10 cells analyzed each time. Scale bars: 10 μm. Two-sample *t*-test (*p < 0.05, **p < 0.01, ***p < 0.001), error bars: SEM.

All proteomic approaches collectively demonstrated altered levels of proteins building cellular vesicles or regulating intracellular vesicular trafficking. Therefore, our next goal was to assess a state of vesicles using the functional assay based on the labeling of neuronal vesicles using Rab7, a vesicular protein commonly occurring in late endosomes, lysosomes, and other types of vesicles that are undergoing active transport in axons and dendrites. Therefore we transfected monomeric red fluorescent protein (mRFP)-tagged Rab7 to SCA3 primary cerebellar neurons (neuron cultures isolated from three different sets of P5 pups, five independent transfections, with 5–10 neurons analyzed each time; Figure 8A).

We found that the average area of vesicles labeled with fluorescently tagged Rab7 was significantly (*t*-test, *p* < 0.01) increased by 40% in Ki91 as compared to WT neurons (Figure 8B). Distribution of vesicle size showed that Ki91 neurons had a lower number of small vesicles (<0.25 μm^2^) and a higher number of large vesicles (>0.5 μm^2^) (*t*-test, *p* < 0.01; Figure 8C). We did not detect any differences in the shape of vesicles. However, there was a significant increase in the number of vesicles normalized to the length of the measured axon (*t*-test, *p* < 0.05; Figure 8D). To assess whether Rab7-loaded vesicles accumulate in either the axon’s proximal or distal region, we also calculated the number of vesicles in those regions, arbitrarily defined as 1/8 of the axon length starting either from the axon hillock or axon tip. There was a significant increase in the number of vesicles in the proximal region (*t*-test, *p* < 0.01) by 58% in Ki91 as compared to WT neurons and in the distal part (*t*-test, *p* < 0.001) by 110% (Figure 8D).

## Ki91 SCA3/MJD Neurons Show Increased Phosphorylation of Axon-Specific Neurofilaments, Indicating the Stress of the Axonal Cytoskeleton

**FIGURE 9:**
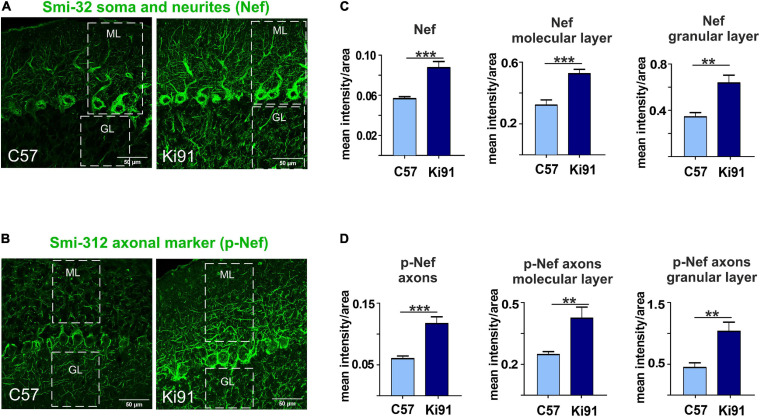
Accumulation of neurofilaments in the cerebellar neurons of Ki91 SCA3/MJD brain. Immunofluorescent staining of the cerebellar sections from 12-month-old mice evaluates the condition of neuronal cytoskeleton and phosphorylation state of neurofilaments in axons. The staining shows the accumulation of phosphorylated neurofilaments in SCA3 cerebellar axons, which is often a sign of axonal stress. Also, the non-phosphorylated pan-neuronal neurofilaments also show increased staining intensity indicating altered cytoskeleton in SCA3 neurons. Scale bars: 50 μm **(A,B)**. Non-phosphorylated heavy neurofilaments (Nef) detected with SMI-32 antibody (pan-neuronal) are presented in panel **(A)**. Phosphorylated neurofilaments H and M detected with SMI-312 (p-Nef; axon-specific) are shown in **(B)**. White boxes inside pictures **(A,B)** mark the area, which was used for measuring the mean intensity of the fluorescence comprising the molecular and granular layer of the cerebellum. Measurement of mean intensity of fluorescence using ImageJ and statistics (*p* < 0.05; two-sample *t*-test) performed for sections as a whole and for both layers separately is presented on the graphs **(C,D)**. Axon-specific SMI-312-positive neurofilaments were highly phosphorylated in both layers in the cerebellum of the Ki91 animals (molecular layer, *p* < 0.001; granular layer, *p* < 0.01; two-sample *t*-test) **(B,D)**. Moreover, the level of dephosphorylated neurofilaments was also elevated in both cerebellar layers of the Ki91 (*p* < 0.01; two-sample *t*-test) **(A,C)**. *N* = 3 biological replicates and 10 confocal images per genotype were collected. Two-sample t-test (*p < 0.05, **p < 0.01, ***p < 0.001), error bars: SEM.

The proteomic approaches and, in particular, the axonal proteomics demonstrated that several classes of dysregulated proteins in SCA3 are located in the axonal compartment. We also found the ataxin-3-positive inclusions along axons in the cerebellum by co-staining with axon-specific marker smi-312 antibody (axon-specific p-Nef). The phosphorylation state of axonal neurofilaments reflects the health of the neuronal cytoskeleton. Therefore we further explored whether the structure and phosphorylation status of neurofilaments are disturbed in the SCA3 model. We assessed the phosphorylated and non-phosphorylated neurofilaments (axon-specific p-Nef and neuronal Nef; Figure 9) on immunostained sagittal brain slices from 12-month-old Ki91 and WT mice using. Increased phosphorylation of neurofilaments is often a sign of defective transport and maintenance also related to mitochondria. We found that both p-Nef levels (SMI-312 antibody) and non-phosphorylated Nef levels (SMI-32 antibody) were significantly increased in the cerebellum of SCA3 mice compared to WT animals (*t*-test, *p* < 0.001; *n* = 3 and five pictures per genotype; Figures 9A–D). In detail, we found increased levels of Nef in soma, dendrites, and axons of Purkinje cells (*t*-test, *p* < 0.01; Figures 9A,C). The increased level of axon-specific p-Nef was detected in axons of the molecular layer and axons of the granular layer and white matter (*t*-test, *p* < 0.001; Figures 9B,D). The altered phosphorylation of axons in the SCA3 Ki91 model may be related to disturbed protein and mitochondria localization and their transport in axons.

## Discussion

Very little is known about the proteome in the SCA3 brain, and it may be one of the reasons for the incomplete understanding of the disease mechanism and delay in finding a cure. Although there are transcriptomic alterations in symptomatic SCA3 animal models, knowledge about changes in levels of the proteins is still missing (Toonen et al., 2018; Zeng et al., 2018). Moreover, we previously demonstrated the lack of transcriptomic changes in the pre-symptomatic SCA3 mice (Wiatr et al., 2019). Here, we aimed to investigate the brain profile of altered proteins in the SCA3 knock-in model to unveil a consensus set of most critical molecular programs governing the disease and identify protein biomarkers. In detail, we aimed to investigate the proteome using three different approaches: at the *in vivo* level, at the CC level, and in relation to disease progression and severity. Once the consensus set of altered candidate processes was established, we aimed to test if we can identify their physiological fingerprints by simple, functional assays, preferably using primary presymptomatic neurons.

We first performed a longitudinal behavioral study between 4 and 18 months of age of Ki91 (Switonski et al., 2015) to establish SCA3 behavioral and phenotypic milestones suitable for the investigation of brain proteome. The first milestone in symptomatic Ki91 mice was the reduced body weight gain identified in 4-month-old animals. Statistically significant gait ataxia at 12-month-old Ki91 was the second milestone, and the last milestone was the severe motor disturbances and progression of other symptoms in 18-month-old animals. Altogether, homozygous Ki91 represents a model of SCA3 progression and gradation of symptoms observed in SCA3 patients (Coutinho and Andrade, 1978; Jacobi et al., 2015; Pulido-Valdeolivas et al., 2016; Coarelli et al., 2018).

In the next step, we determined a range of affected brain areas by the MRI and detection of ataxin-3-positive inclusions using advanced, symptomatic Ki91 animals. The inclusions were present in almost all brain areas of both the posterior and frontal brain parts. The intra-nuclear inclusions dominated in the cerebellum, mainly in the white matter, DCN, and in pons and midbrain; however, they were also present in the cerebral cortex, hippocampus, and striatum. Importantly, we have seen highly abundant ataxin-3-positive inclusions in SMI-312-positive cerebellar axons in white matter. The intra-axonal inclusions were also previously detected in nerves and various tracts in SCA3/MJD patients (Seidel et al., 2010). Also, the changes observed by brain MRI in Ki91 were not localized to one structure, but the atrophy was spreading on several brain regions, including frontal parts, cerebellum, and brainstem. Moreover, brain areas rich in axons such as the corpus callosum are atrophied in Ki91. Therefore, exploring our behavioral, neuropathologic, and MRI results led to selecting the consensus set of brain regions for further experiments. To identify molecular SCA3 pathogenesis by our three-way proteomic approach, we selected two representative brain regions: the cerebellum and cerebellar cortex. Both regions demonstrated a high number of inclusions; particularly, the cerebellum was affected more than other brain regions. Besides, the cerebral cortex demonstrated very pronounced atrophy on MRI. Even if it was not possible to determine cerebellar volume change on MRI (technical limitation of MRI setup), our previous works detected atrophy of Purkinje cells and occurrence of reactive gliosis by immunohistochemistry (Switonski et al., 2015). Also, the behavioral motor signs indicated the involvement of cerebellum atrophy. Such a selection of brain regions for further experiments involves both classically reported SCA3 pathogenesis in the hindbrain and postulated SCA3 pathogenesis in more frontal parts of the brain (Schmidt et al., 2019; Guo J. et al., 2020).

Our first proteomic approach corresponded to Ki91 behavioral milestones where we selected tissue from animals at the age of 4 months (lack of weight gain), 10 months (mice on the verge of behavioral phenotype), 12 months (motor decline statistically significant), and 14 months (an additional symptomatic age). The second proteomic approach, called “correlative,” was aimed to identify dysregulated proteins and processes in mouse subjects with carefully characterized and matching phenotype severity. The first two proteomic approaches offered insights into the disease pathogenesis at the level of tissue and disease stage; however, the perspective of cellular level and CCs were still missing in both approaches. Therefore we employed a third, targeted proteomics approach where we separately assessed proteins in somatodendritic and axonal compartments of primary neurons isolated from embryos or pups. An additional advantage of such primary neuronal SCA3 culture is the possibility of validation if processes discovered in two other approaches may occur early in the disease development.

The first pathogenic process, which was consensus among the proteomic approaches in our work, is the disturbed energy metabolism. We demonstrated many dysregulated proteins related to the mitochondrial compartment, which corresponds with the reduced body weight gain in Ki91. Notably, the BMI of SCA3 patients is significantly lower, and BMI decrease correlated with faster disease progression (Diallo et al., 2017; Yang et al., 2018). The energy metabolism was the most common pathway, and the proteins related to metabolism were dysregulated already in 4-month-old presymptomatic Ki91 mice. For instance, many OXPHOS-related proteins, such as Nduf subunits, Aco2, and mt-Co proteins and several metabolic proteins such as Gpd2, Cs, Gpi, Pkm, Wdr1, Sdha, Ddx1, and Aldh1l1 demonstrated altered levels and were previously identified in AD, HD, and SCA1 (Sorolla et al., 2008; Sánchez et al., 2016; Lunnon et al., 2017). Similarly, the correlative approach also revealed downregulated mitochondrial proteins such as mitochondrial chaperone Timm9, Ndufb5, and Fasn in both “mild” and ‘severe” phenotype, which suggest that disturbances of energy production is the primary pathology of SCA3. Several mitochondrial proteins were also depleted in axons compared to the somatodendritic compartment in SCA3 neurons, which may indicate the disrupted delivery of mitochondria to nerve terminals. The most frequently dysregulated protein, which we propose as one of a SCA3 biomarker candidate in both tested brain regions, is carbonic anhydrase 2 (Ca2). Ca2 is linked to a mechanism controlling an increased transport of lactate, which is an important energy source for neurons (Stridh et al., 2012; Karus et al., 2015).

Since many proteomic changes pointed out pathways related to energy metabolism, our first functional assay measured mitochondrial respiration and glycolysis rate in cultured cerebellar Ki91 neurons kept for DIV 3, 11, 18, and 21, which reflected the culture differentiating from young to more mature neurons. Commonly, the typical culture of such neurons and also our WT neurons represented a developing dependency on OXPHOS since mature neurons rely on OXPHOS to meet energy demands. In turn, SCA3 neurons demonstrated a lack of increase in OXPHOS dependency and even a slight drop in oxygen consumption during neuronal maturation. SCA3 neurons under stress reached their maximal OCR already as young as DIV 11 as compared to WT neurons, which reached the maximum at DIV 21. At DIV 3 and 11, SCA3 neuronal progenitors show higher overall oxygen consumption; therefore, at this early stage, the neurons may already be in deficiency of energy, or its production is aberrant. In the case of older SCA3 neurons with highly developed processes, a dramatic difference in energy metabolism compared to WT neurons can also be related to the failure of energy production in axons.

Overall, we demonstrated that the number of dysregulated proteins related to metabolism, lysosomes, endosomes, and protein folding increases with Ki91 age and is noticeably higher in older 10–14-month-old Ki91 mice, which represent the phase of the motor symptoms development. Moreover, the most advanced disease stage marked by highly reduced body weight, anxiety, and severe motor symptoms in 18-month-old animals is also characterized by the excessive size of Atxn3 inclusions and were observed both in the nuclei and axons, whereas in pre-symptomatic 2-month old SCA3 mice ataxin-3-positive aggregates were relatively smaller and detected only in cell nuclei (Wiatr et al., 2019).

Recent discoveries emphasize the role of degeneration of “wiring” between brain structures and axonal deficits as one of the main pathogenic events in neurodegenerative disorders (Sánchez-Valle et al., 2016; Ferrari Bardile et al., 2019; Casella et al., 2020). Also, in SCA3, the axonal defects and white matter abnormalities are prominent disease symptoms (de Rezende et al., 2015; Farrar et al., 2016; Lu et al., 2017). Our proteomic approaches jointly demonstrated that the largest population of proteins with altered levels was involved in vesicular transport and endocytosis as a second consensus molecular process candidate in SCA3. Vesicular trafficking is crucial for axon maintenance, synaptic signaling, releasing, and recycling of neurotransmitters. Our proteomic data demonstrate highly downregulated levels of proteins related to vesicle transport or trans-synaptic signaling (Nrgn, Sh3gl1, Vamp2, Syngap1, Bsn, Syt7, Homer1, Rims1, Pclo, and Erc2; SNARE complex). Several vesicular Rab proteins were also dysregulated, such as Rab11b, Rab3d, and Rab10. Other Rab proteins were also dysregulated in SCA3/MJD patients (Sittler et al., 2018). Besides, there were dysregulated proteins related to protein metabolism localized to endosomes, exosomes, and lysosomes. Many of these proteins are involved in neurodegenerative diseases such as AD and HD (Skotte et al., 2018; Mendonça et al., 2019), and their loss brings significant neurological consequences (Yoo et al., 2001; Santos et al., 2017; Salpietro et al., 2019; Falck et al., 2020). Moreover, Qdpr (human homolog: DHPR) and Glul proteins responsible for neurotransmitters’ metabolism are progressively downregulated throughout the SCA3 course in the Ki91 cerebellum and cortex. Both proteins are essential for proper CNS function (Breuer et al., 2019; Kurosaki et al., 2019; Zhou et al., 2019).

The 3rd consensus SCA3 candidate process was termed by us “the regulation of cytoskeletal structure and function” with dysregulated proteins localizing to neuronal processes. Coherently with vesicle trafficking, our proteomic results also revealed dysregulation of neurofilament proteins (NFs), such as heavy chain NF-H in both the cerebral cortex and cerebellum. Disturbed energy metabolism in neurons is often related to defects in the cellular cytoskeleton and phosphorylation state of NF-H, in particular, resulting in aberrant axonal transport of mitochondria (Jung et al., 2000; Shea and Chan, 2008). Furthermore, proteomic data contained dysregulated proteins pivotal for microtubular cytoskeleton stability and axon organization. Dysregulated proteins were also involved in anterograde and retrograde microtubule-based transport (Kif5c, Kif5b, Kif21a, Klc1, Klc2, and Kif3a; Dnm3, Dync1li1, Dync1h1, and Dynll2), in particular in “severe” phenotype, which may suggest that disruption of axonal transport contributes to increased severity of SCA3 symptoms. Among the microtubular proteins, Tau (Gao et al., 2018) was elevated in the cerebral cortex of symptomatic Ki91 mice, RhoG (Zinn et al., 2019), and dysregulated group of proteins that are also enriched in oligodendrocytes such as Cryab protein stabilizing cytoskeleton and previously proposed as a component of therapeutic strategy (Ghosh et al., 2007; Houck and Clark, 2010; Cox and Ecroyd, 2017; Zhu and Reiser, 2018). Dysregulation of Cryab, RhoG, Mbp, and Marcks indicates that oligodendrocytic processes and myelination contribute to axon dysfunction in SCA3 (Ishikawa et al., 2002; Guimarães et al., 2013; Zhang et al., 2017; Rezende et al., 2018). In addition, we previously identified altered levels of mRNA related to oligodendrocyte differentiation and function in older SCA3 mice (Wiatr et al., 2019). Interestingly, several oligodendrocytic marker proteins dysregulated in our proteomic studies, such as Plp, Qdpr, Mbp, and Mog were predicted to localize in extracellular vesicles (EVs). EVs are often released by oligodendrocytes and deliver trophic support for axons but may also transfer pathogenic triggers (Krämer-Albers et al., 2007; Porro et al., 2019).

The investigation of two candidate consensus processes related to vesicle transport and neuronal cytoskeleton required a targeted proteomic approach focused on axons. The proteins that were deficient in SCA3 axons were associated with vesicles, axonal transport, and mitochondria. Depleted proteins suggesting altered transport along the axon in Ki91 included proteins interacting or forming dynactin complex, required for mitochondria and endosome/lysosome and synaptic vesicles transport along the axons, such as Actr1a and Ank2 (Holleran et al., 1996; Moughamian et al., 2013; Lorenzo et al., 2014). Besides, there were also essential cytoskeletal proteins involved in regulating axonal transport and actin dynamics, Arf6 and its interactor RhoB, targeted by Arf6 to endosomes (Eva et al., 2017; Zaoui et al., 2019). Components of SNAREs responsible for vesicular trafficking were also depleted in SCA3 axons, such as Napa (a.k.a. α-SNAP), Vti1b, and Nsf. Lower levels of Napa, Nsf, and Actr1a were also identified by the “parallel” proteomic approach in the cerebellum of Ki91 mice. Of note, Nsf is the ATPase indispensable for membrane fusion and for regenerating active SNAREs. Nsf depletion results in the arrest of membrane trafficking and, consequently, neuronal damage and death (Stenbeck, 1998; Emperador-Melero et al., 2018; Yuan et al., 2018). In particular, disturbed axonal transport may affect delivery and proper localization of mitochondria since we detected several essential mitochondrial proteins with diminished levels in Ki91 axons.

In addition to defective energy metabolism, all of our proteomic approaches identified the vesicular processes and cytoskeletal structure and regulation processes in the SCA3 Ki91 brain. In addition to vesicular and cytoskeletal localization, most dysregulated proteins identified in proteomics were predicted to localize in axons. Therefore we designed two simple assays to detect functional fingerprints of vesicular and cytoskeletal phenotypes in neurons. We decided to load the neuronal vesicles with fluorescent Rab7 protein as the universal, external marker often occurring in a variety of vesicle species. We were able to detect the accumulation of vesicles in cell bodies and in the processes of SCA3 neurons. Moreover, the vesicles were enlarged in SCA3 neuronal processes. The second assay was based on immunostaining detection of axonal cytoskeleton phosphorylation to assess a putative pathogenic process related to the axonal cytoskeleton. We used the SMI-312 antibody, an axon-specific marker (phosphorylated NFs), and detected highly increased phosphorylation in the 12-month-old Ki91 cerebellum compared to WT cerebellum. Non-phosphorylated NFs (SMI-32; pan-neuronal staining) were also increased in the SCA3 KI91 cerebellum. Accumulation and aggregation of phosphorylated neurofilaments in axons is a biomarker indicating axonal damage, which may disturb the transport of vesicles and mitochondria. Moreover, in neurodegenerative disorders and SCA3, the NFs could serve as a biomarker (Li et al., 2019).

Lastly, axonal proteomics in Ki91 has also demonstrated highly enriched protein translation machinery represented by ribosomal proteins, various RNA-binding proteins, and translation initiation factors (Supplementary Tables 5, 7). Protein synthetic machinery is intensively transported to the axons in response to cellular stress (Baleriola and Hengst, 2015). We detected the signs of cellular stress by identifying elevated Tpp1 and Tmem33 proteins, which play a role in unfolded protein response (UPR) (Sakabe et al., 2015; Mukherjee et al., 2019). Along this line, protein chaperons and proteasome subunits (Cct2 and Pmsd14) were highly enriched in SCA3 Ki91 axons. Moreover, ribosomal and RNA-binding proteins are also compounds of RNP granules (mRNPs, messenger ribonucleoproteins) or stress granules, both holding mRNAs translationally repressed (Sossin and DesGroseillers, 2006; El Fatimy et al., 2016). One of the crucial components of the granules is Fxr2 (Chyung et al., 2018), which is highly enriched in Ki91 cerebellar axons. Interestingly, vesicular platforms in axons were recently identified as carriers of translation machinery, mRNPs, and structure responsible for axonal mitochondria maintenance (Cioni et al., 2019). Furthermore, it was also demonstrated that protein inclusions specifically impair the transport of endosomes and autophagosomes (Volpicelli-Daley et al., 2014; Guo W. et al., 2020). Also, locally active translation machinery and local energy production are long known phenomena in axons since it provides critical substrates even to the most distal part of the neuronal terminals. However, in the case of accumulation of the polyQ proteins, such an organization of protein synthesis in long processes may accelerate neuronal dysfunction. In such a case, upregulated translation machinery may be a “vicious cycle” for the accumulation of polyQ proteins in axons, gradually aggravating the disease and leading to profound neuronal dysfunction.

## Conclusion

**FIGURE 10:**
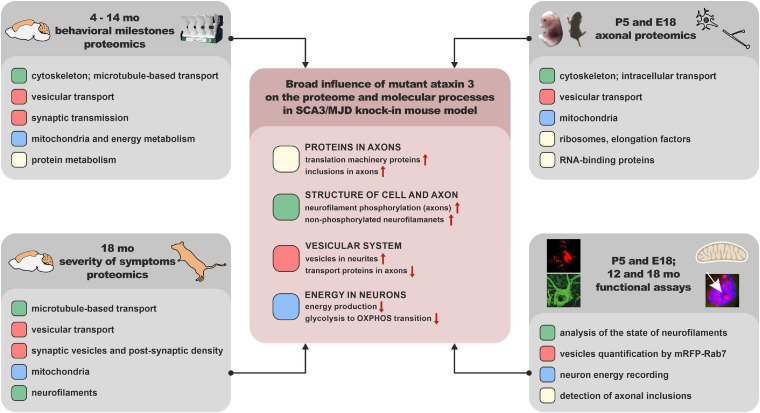
The graphical summary of broad influence of mutant ataxin 3 on the proteome and molecular processes in SCA3/MJD knock-in mouse model.

Spinocerebellar ataxia type 3 molecular disease mechanism is insufficiently explored. Ataxin-3 is ubiquitin protease, but very little is known about the broader influence of mutant protein on other proteins in the SCA3 brain. The missing knowledge about the proteome may be one of the reasons for the incomplete understanding of the disease mechanism and lack of cure. Therefore, we present the first global model picture of SCA3 disease progression based on the alterations in protein levels in the brain and embryonic SCA3 axons, combined with an *in vivo* behavioral study and neuropathology. We propose the most relevant pathogenic processes in SCA3 involving disturbed energy metabolism, impairment of the vesicular system, cell and axon structure, and altered protein homeostasis. We demonstrate that the consensus processes identified in the SCA3 Ki91 brains show their functional signs in the primary pre-symptomatic neurons, and therefore may be the first steps of pathogenesis. Our data have demonstrated for the first time that one of the essential pathogenic aspects of SCA3 is the faulty localization of proteins between axons and soma. Further signs of affected SCA3 axons are vesicle accumulation and increased neurofilament phosphorylation. The important novel finding is the highly increased axonal localization of proteins involved in the translation machinery in SCA3. It is possible that local mutant protein production in axons may accelerate neuronal dysfunction; therefore, upregulated translation machinery in axons may aggravate the disease. Moreover, we identified a variety of molecular signatures related to mitochondria in the SCA3/MJD Ki91 model, and we show that the earliest global process which occurs already in the neonatal neurons is the OXPHOS and glycolysis deficits. For the first time, we demonstrate that the young developing SCA3 neurons fail to undergo the transition from glycolytic to OXPHOS phenotype typical for normal neurons. Summarizing, we revealed that the SCA3 disease mechanism is related to the broad influence of mutant ataxin-3 on the brain, neuronal and axonal proteome, and disruption of the proper localization of axonal and somatodendritic proteins and organelles such as vesicles, translation machinery, and mitochondria (Figure 10).

## Materials and Methods

### Animals

Maintaining and breeding were performed at standard conditions with an 18/6-h light/dark cycle and water and food *ad libitum*. No differences in food or water intake between Ki91 and WT mice were observed. The animals were marked using numerical ear tags (National Band & Tag Company, Newport, RI, United States). The stress level of the animals was minimized throughout all procedures and animal handling. The animal experimentation and handling were approved and monitored by the Local Ethical Commission for Animal Experiments in Poznań. The animals were sacrificed according to AVMA Guidelines for the Euthanasia of Animals by placing them in the programmable CO_2_ chamber (Rothacher Medical, Heitenried, Switzerland). Homozygous SCA3 Ki91 animals contained between 103 and 132 CAG repeats on a single mutant ataxin3 allele. SCA3 Ki91 mice (C57BL/6 background) and age-matched WT littermates bred in-house were used for behavioral tests (*n* = 36) and collecting brain tissues for proteomic analysis (*n* = 32, and *n* = 21, after behavioral tests), validation of proteomics (*n* = 32), MRI (*n* = 12) and immunostaining (*n* = 8, after behavioral tests and *n* = 6 from a separate cohort of 12-month-old animals). The cerebellum and cerebral cortex for proteomic analysis were collected from 4, 10, 12, 14, and 18 month-old animals, which corresponded to the timepoints of behavioral testing. An experimental group consisted of four mutant mice vs. four WT littermates (proteomics: 4, 10, 12, and 14-month-old and immunostaining), and 11 mutant mice vs. 10 WT littermates (proteomics: 18-month-old).

### Behavioral Studies

Mice were trained and tested for motor deficits, starting from 4-month-old animals, and consequently every 2 months, until the age of 18-month-old in a cohort of 18 mutant mice vs. 18 WT littermates. Tests included accelerating rotarod (4–40 rpm in 9.5 min), elevated beam walk (diameter of rods: 35, 28, 21, 17, and 9 mm) as previously described (Switonski et al., 2015), and additionally in 18-month-old animals open field test in which locomotor activity and zone preferences in the experimental cage (50 cm^2^) were analyzed and measured during 10 min. Each test consisted of 1 training day (T) and 3 consecutive days of measurement. We also performed scoring tests designed for evaluation of the ataxia phenotype in mouse models (Guyenet et al., 2010), wire test for evaluating muscle strength, and footprint for gait ataxia. For gait analysis, the forelimbs of the mice were stained with black and hindlimbs with red non-toxic paint. Mice were placed on a sheet of paper inside a tunnel (80 × 10 cm). Only the middle part of a series of steps was measured for the distance between two steps (stride length), for- and hindlimb base, and overlapping between for- and hindlimb. All mice were weighed during each testing session. Graphing and statistics were performed on PrismVR software (San Diego, CA, United States), using ANOVA with Bonferroni *post hoc* test.

### Magnetic Resonance Imaging and Image Analysis

After PFA perfusion of the mice, brains (*n* = 6 per genotype) were removed and stained by a one-week soaking in Gadolinium solution (Dotarem, Guerbet, France) in PBS at 2.5 mM. This protocol enhances the signal- and contrast-to-noise ratios on MR images of fixed brains (Dhenain et al., 2006). *Ex vivo* MRI experiments were performed on a horizontal 11.7 T Bruker scanner (Bruker, Ettlingen, Germany). A quadrature cryoprobe (Bruker, Ettlingen, Germany) was used for radiofrequency transmission and reception. Diffusion Tensor Imaging (DTI) data were acquired using Echo Planar Imaging sequence (Resolution = 100 × 100 × 200 μm3, TR = 3,000 ms, TE = 28 ms, 60 directions). To preserve their integrity and avoid deformations, brains were kept inside skulls for *ex vivo* MRI experiments. Anatomical images were co-registered in the same space to create a study template. Transformations to match the study template to an atlas composed of a high-resolution template and a map of region’s labels adapted from the Allen mouse brain Atlas (Lein et al., 2007) were calculated. Finally, transformations to match the study template and labels to each anatomic image were calculated. The automated segmentation pipeline was performed using an in-house python library (Sammba-MRI^1^).

### Protein Sample Preparation

Protein extraction was performed as previously described (Wiatr et al., 2019). Mouse brain tissues were homogenized with a Mixer Mill MM400 (Retch, Haan, Germany), followed by a threefold cycle of freezing and thawing and bath sonication (3 × 3-min). Protein concentration was estimated using Quant kit (GE Healthcare, Chicago, IL, United States). Ten-microgram aliquots of proteins were diluted with 15 μl of 50 mM NH4HCO3 and reduced with 5.6 mM DTT for 5 min at 95°C. Samples were then alkylated with 5 mM iodoacetamide for 20 min in the dark at RT. Subsequently, the proteins were digested with 0.2 μg of sequencing-grade trypsin (Promega, Madison, WI, United States) overnight at 37°C.

### Liquid Chromatography Coupled to Tandem Mass Spectrometry

The analysis of the proteome was performed with the use of Dionex UltiMate 3000 RSLC nanoLC system connected to the Q Exactive Orbitrap mass spectrometer (Thermo Fisher Scientific, Waltham, MA, United States). Peptides derived from in-solution digestion were separated on a reverse-phase Acclaim PepMap RSLC nanoViper C18 column (75 μm × 25 cm, 2 μm granulation) using acetonitrile gradient (from 4 to 60%, in 0.1% formic acid) at 30°C and a flow rate of 300nL/min (for 230 min). The spectrometer was operated in data-dependent MS/MS mode with survey scans acquired at a resolution of 70,000 at m/z 200 in MS mode and 17,500 at m/z 200 in MS2 mode. Spectra were recorded in the scanning range of 300–2,000 m/z in the positive ion mode. Higher energy collisional dissociation (HCD) ion fragmentation was performed with normalized collision energies set to 25. Swiss-Prot mouse database was used for protein identification with a precision tolerance set to 10 ppm for peptide masses and 0.08 Da for fragment ion masses. Raw data obtained for each dataset were processed by MaxQuant 1.5.3.30 version for protein identification and quantification. Protein was considered as successfully identified if the Andromeda search engine found at least two peptides per protein, and a peptide score reached the significance threshold FDR = 0.01.

Obtained data were exported to Perseus software ver. 1.6.1.3 (part of MaxQuant package; Munich, Germany). Numeric data were transformed to a logarithmic scale, and each sample was annotated with its group affiliation. Proteins only identified by site, reverse database hits, and contaminants were removed from the results. Next, data were filtered based on valid values for proteins. Proteins that contained valid values in 75% of samples in at least one group were included as valid hits. Before statistical analysis, normalization of data was performed by subtracting the median from each value in a row. A two-sample *t*-test was performed on analyzed sample data with *p*-value < 0.05 was considered as significant, and differentiating proteins were normalized using the *Z*-score algorithm for the hierarchical clustering of data.

### Bioinformatic Analysis of Proteomic Data

Proteins were grouped using the CPDB according to the pathways (pathway enrichment *p*-value cut-off < 0.01), GO term by molecular function (MF), biological process (BP), and cellular component (GO term B, MF level 5, CC level 4 and 5, *p*-value cut-off < 0.01). CPDB integrates data from 30 different resources, including such databases as Reactome, KEGG, and BioCarta (Kamburov et al., 2013). Next, common pathways and GO terms for four datasets separately in both tissues (4, 10, 12, and 14 months of age) containing the highest relative number of dysregulated proteins and *p*-values were selected. The relative number of dysregulated proteins was calculated as a number of dysregulated proteins involved in the pathway or GO term divided by the total number of dysregulated proteins in a given dataset (%). For each analysis, the names of genes corresponding to the names of dysregulated proteins were used. In the separate analysis, we performed identification of cell types in the brain, which are affected by pathogenesis with the use of the Dropviz tool (Saunders et al., 2018) for the cerebellum and cerebral cortex. Significantly dysregulated (*p* < 0.05) proteins identified from label-free proteomic and experiments were included together into two tissue groups based on origin from the cerebellum and cortex. Relative expression is presented in Dropviz as the number of transcripts per 100,000 in the cluster. The analysis of proteins displaying altered ratio between axon and soma in cerebellar and cortical neurons of Ki91 mice was performed using the String database^2^ and clustering tool (MCL clustering, inflation parameter = 6).

### Vacuum Dot Immunoblot

Before use in the dot blot assay, each antibody was validated with western blot assay (WB). WB was performed as previously described (Wiatr et al., 2019). Cerebellum and cortex samples were harvested from 16 homozygous Ki91 animals and 16 WT littermates; *n* = 4 mice per tested age (4, 10, 14, and 18 months of age) and per each genotype. The protein concentration was estimated using a Pierce BCA protein assay kit (Thermo Fisher Scientific, Waltham, MA, United States). The nitrocellulose membrane and Whatman filters (all GE Healthcare, Chicago, IL, United States) were washed two times with Milli-Q H_2_O and two times with Tris-Buffered Saline (TBS) by vacuum filtration. A total of 2.5 μg of each protein sample in 50 μL of PB lysis buffer with PMSF cocktail inhibitor (Sigma-Aldrich, St. Louis, MO, United States) was spotted on nitrocellulose membrane and washed two times with TBS by vacuum filtration, and then allowed to air-dry. The blots were stained with Ponceau S solution and blocked with 5% non-fat milk in PBS/0.05% Tween-20 for 1 h at RT and subsequently incubated at 4°C overnight with the following primary antibodies: rabbit anti-MBP (1:1,000; Cell Signaling, Danvers, MA, United States), mouse anti-CRYAB (1:1,000; Developmental Studies Hybridoma Bank, Iowa City, IA, United States), mouse anti-GLUL (1:1,000; BioLegend, San Diego, CA, United States), rabbit anti-CA2 (1:2,000; ProteinTech, Rosemont, IL, United States), rabbit anti-QDPR (1:2,000; ProteinTech, Rosemont, IL, United States). The blots were probed with the respective HRP-conjugated secondary antibody (anti-rabbit or anti-mouse, 1:2,000; Jackson Immuno Research, Suffolk, United Kingdom). The immunoreaction was detected using the ECL substrate (Thermo Fisher Scientific, Waltham, MA, United States).

### Primary Neuron Culture and Transfection

Primary neuron cultures were derived from Ki91 and C57 mice according to AVMA Guidelines for the Euthanasia of Animals. Cortices were dissected from E18 mouse embryos and cerebella from P5 pups. Cortical neurons were dissociated by trypsin (Merck, Darmstadt, Germany) diluted 10×, and cerebellar neurons were dissociated by trypsin diluted 5×. Cells were washed two times in a disassociation medium consisting of HBSS (Thermo Fisher Scientific, Waltham, MA, United States), supplemented with 0.1% glucose, 1× penicillin-streptomycin (Thermo Fisher Scientific, Waltham, MA, United States), then two times in a plating medium consisting of Dulbecco’s modified Eagle medium (DMEM), 1× CTS GlutaMAX-I supplement, 1× penicillin-streptomycin (all Thermo Fisher Scientific, Waltham, MA, United States). Clumps of tissue debris were allowed to settle to the bottom of a 15 ml tube, and the supernatant containing dissociated cells was centrifuged for 3 min at 1,300 rpm. Cells were seeded onto coverslips, tissue culture inserts (Greiner Bio-One GmbH, Kremsmünster, Austria), or Seahorse XFp Cell Culture Miniplates (Agilent Technologies, Santa Clara, CA, United States) coated with poly-D-lysine (Thermo Fisher Scientific, Waltham, MA, United States). Neurons were maintained in conditioned Neurobasal medium (Thermo Fisher Scientific, Waltham, MA, United States) supplemented with 2% B27 supplement, 1× CTS GlutaMAX-I supplement, 1× penicillin-streptomycin (all Thermo Fisher Scientific, Waltham, MA, United States), 1× apo-transferrin (Sigma-Aldrich, St. Louis, MO, United States), and N2 (0.005 mg/ml insulin, 0.0161 mg/ml putrescin, 30 nM Na-Selenite, 51 nM T3, 20 nM progesterone) in a humidified incubator with 5% O_2_ and 5% CO_2_ in air at 37°C. The maintenance medium was replenished with half-feed changes every 2–3 days. For transfection, cells were seeded at a density of 2 × 10^5^ cells/well onto coverslips in 24-well culture plates. Neurons were transfected at DIV 3–4 with mRFP-Rab7 (Addgene plasmid # 14436^3^; dgene_14436) at a concentration of 0.8 μg DNA per well using Lipofectamine 2000 (Thermo Fisher Scientific, Waltham, MA, United States) followed by fixation (4% PFA, 15 min in RT) and confocal imaging at DIV 7–11 from three biological replicates and five independent transfections. An approximately 10–15% transfection efficiency was achieved.

### Isolation of Axons

Dissociated neurons (cortical and cerebellar) were seeded at a density of 1 × 10^6^ cells/well onto tissue culture inserts containing porous membrane (1 μm pores; Greiner Bio-One GmbH, Kremsmünster, Austria) in a 6-well cell culture plates. The bottom compartment medium was supplemented with 15 ng/ml of BDNF (PeproTech, London, United Kingdom) so that it could act as a chemoattractant for axons growing through the insert membrane. Axonal fraction and the fraction containing neuronal body with dendrites were isolated by carefully detaching the cellular contents from the inner and outer insert membrane surface with a cell scraper in TAEB buffer (Merck, Darmstadt, Germany) four times, alternating the direction by 90° each time. Axons were collected after 11 days in culture because, after DIV14, dendrites, which grow at a slower rate, are also detected on the other side of the porous membrane. Before isolation of the axon part, the inner membrane surface was scrubbed with a cotton-tipped applicator, and the membranes were cut out from the insert. To examine whether the separation of axons from the somatodendritic part was correctly performed, we also immunostained culture filters isolated from the Boyden chamber before and after scraping off the cell bodies, with immunofluorescence assay (β-III-tubulin, 1:500; Burlington, MA, United States) and nuclear dye Hoechst (Supplementary Figures 5A,B). The presence of axons on the outer site of the membrane was examined, and the purity of the obtained axonal fraction was evaluated with nuclear and axonal markers (Supplementary Table 3). The axon isolation procedures were adapted from Unsain et al. (2014).

### Seahorse XFp Cell Energy Phenotype Test

Seahorse XFp Cell Energy Phenotype assays (Seahorse, Agilent Technologies, Santa Clara, CA, United States) were performed according to the manufacturer’s instructions on an XFp instrument. Dissociated cerebellar neurons were seeded at a density of 5,300 or 12,500 cells/well onto culture mini plates with the protocol described in the section “Primary neuron culture and transfection.” The assays were performed at DIV3, 11, 18, and 21. On the day of assay, the XF assay medium was supplemented with 25 mM glucose, 0.5 mM pyruvate, and 2 mM glutamine, and the pH adjusted to 7.4. After assay performance, cells were lysed with PB1× buffer, and protein concentration was measured, and the obtained values were used for data normalization. Data analysis was performed using the Seahorse XFe Wave software and the Seahorse XF Cell Energy Phenotype Test Report Generator. The baseline OCR/ECAR ratio was higher than 4 only in DIV3, which means that the stressed ECAR parameter, in this case, may include both glycolysis and mitochondrial activity.

### Immunofluorescence Staining

The animals were deeply anesthetized and transcardially perfused using saline, followed by 4% PFA. The brains were removed, post-fixed in 4% PFA for 48 h, and cryopreserved with graded sucrose (10–20–30%) over 72 h. The 20 or 30-μm parasagittal mouse brain sections were cut using a cryostat at −20°C and collected on SuperFrost Plus slides (Thermo Fisher Scientific, Waltham, MA, United States). The sections were processed immediately. The HIER procedure was applied by incubation of the sections in citrate buffer (pH 9.0) for 30 min at 60°C. The sections were blocked via incubation in 4% normal goat serum in TBS for 1 h. For immunofluorescent staining, the sections were incubated overnight at 4°C with the primary mouse anti-ataxin-3 antibody 1H9 (1:200; kindly provided by Yvon Trottier), rabbit anti-ataxin-3 (1:200; ProteinTech, Rosemont, IL, United States), rabbit anti-ubiquitin (1:500; Z0458, Dako, Jena, Germany), mouse SMI-32 against non-phosphorylated heavy and medium neurofilament subunits (1:1,000) and mouse SMI-312 against phosphorylated heavy and medium neurofilaments (1:1,000) (Biolegends, San Diego, CA, United States), and subsequently with the anti-mouse or anti-rabbit antibody labeled by AlexaFluor488, Dylight594, or AlexaFluor647 (1:400; Jackson ImmunoResearch; Suffolk, United Kingdom). The sections were end-stained with Hoechst 33342 (Sigma) nuclear stain at 1:1,000 and embedded in Fluoroshield (Sigma) mounting medium.

### Image Acquisition and Quantification

Confocal images were acquired using two microscope systems. The first system was Opera LX (PerkinElmer) using 40× water objective, 200 ms exposure time, and 50–70% laser power. For each picture, 25–50 confocal sections were collected, from which 10 sections were dissected for further analysis. The second system was TCS SP5 II (Leica Microsystems; Poland), using oil immersion 63× objective with a sequential acquisition setting. For fluorescent quantification, images were acquired using the same settings at a resolution of 1024 × 1024 pixels and 100 Hz. Confocal sections (8–10) were acquired to a total Z-stack thickness of 0.13 μm. For each condition, we performed three independent cultures; and five independent transfections from which 46 pictures of Ki91 and 33 pictures of WT were collected and analyzed. For analyzing the state of mrFp-Rab7+ vesicles in fixed neurons, we selected axons, which were distinguished from dendrites based on known morphological characteristics: greater length and sparse branching (Banker and Cowan, 1979). For immunostaining of mouse brains, we used four slices, and randomly acquired from each slice, ≥5 fields were. Offline analysis of the image Z-stack was performed using the open-source image-processing package Fiji/ImageJ. Morphometric measurements were performed using Fiji/ImageJ. Measured data were imported into Excel software for analysis. The thresholds in all images were set to similar levels. Data were obtained from at least three independent experiments, and the number of cells or imaging sections used for quantification is indicated in the figures. All statistical analyses were performed using the Student’s *t*-test and are presented as mean ± SEM.

### Statistical Analysis

The data regarding behavioral experiments were subjected to a two-way ANOVA, followed by Bonferroni pos*t*-tests. *p*-values of less than 0.05 were considered significant. Identification of proteins on raw proteomic data was performed by the Andromeda search engine in Mascot using the following inclusion criteria: at least two different peptides per protein were identified per sample, and a total peptide score reached the significance threshold FDR = 0.01. Identified proteins matching the inclusion criteria were subjected to further statistical analysis with a two-sample *t*-test, and dysregulation of protein level reaching *p*-value < 0.05 was considered as significant. Kruskal–Wallis test was used to perform a score assessment in the 0–5 scale of mild, moderate, and severe phenotype in 18-month-old animals (*p*-value < 0.05).

## Data Availability Statement

The datasets presented in this study can be found in online repositories. The names of the repository/repositories and accession number(s) can be found below: PRIDE, PXD024945.

## Ethics Statement

The animal study was reviewed and approved by the Local Ethical Committee for Animal Experiments in Poznań.

## Author Contributions

MF was responsible for the research concept and obtaining funding, conceived, designed, and supervised all experiments, and analyzed the data. KW and ŁM performed all proteomic experiments. KW analyzed the data, designed and performed all experiments except of MRI. EB, J-BP, and JF performed the MRI experiment and analyzed the data. MF and KW wrote the manuscript. All authors contributed to the article and approved the submitted version.

## Conflict of Interest

The authors declare that the research was conducted in the absence of any commercial or financial relationships that could be construed as a potential conflict of interest.

## Footnotes

1 https://github.com/sammba-mri/sammba-mri

2 https://string-db.org/

3 http://n2t.net/addgene:14436

## Supplementary Material

Supplementary Table 1 MS/MS proteomic data for each tested age (4, 10, 12, and 14-month-old) in the parallel approach in the cerebellum.

Supplementary Table 2 MS/MS proteomic data for each tested age (4, 10, 12, and 14-month-old) in the parallel approach in the cerebral cortex.

Supplementary Table 3 MS/MS proteomic data for 18-month-old Ki91 cerebellum and cortex in the correlative approach.

Supplementary Table 4 MS/MS proteomic data for the of protein levels in somatodendritic and axon compartment in cerebellar and cortical samples.

Supplementary Table 5 Proteins depleted in Ki91 cerebellar axons.

Supplementary Table 6 Proteins enriched in Ki91 cerebellar axons.

Supplementary Table 7 Proteins depleted in Ki91 cortical axons.

Supplementary Table 8 Proteins enriched in Ki91 cortical axons.

## Abbreviations

ANOVA: analysis of variance
BP: biological process
CPDB: ConsensusPath Database
CC: cellular compartment
CNS: central nervous system
DIV: days in vitro
ECAR: extracellular acidification rate
FC: fold change
MJD: Machado-Joseph disease
MS: mass spectrometry
mo: month-old
MF: molecular function
mRFP: monomeric red fluorescent protein
MRI: magnetic resonance imaging
mRNP: messenger ribonucleoprotein
NF: neurofilament
OCR: oxygen consumption rate
OXPHOS: oxidative phosphorylation
polyQ: polyglutamine
PCA: principal component analysis
SCA3: Spinocerebellar ataxia type 3
TCA: tricarboxylic acid cycle
WT: wildtype.
