# Supplementary material for: Broad Influence of Mutant Ataxin-3 on the Proteome of the Adult Brain, Young Neurons, and Axons Reveals Central Molecular Processes and Biomarkers in SCA3/MJD Using Knock-In Mouse Model
Source: Front Mol Neurosci. 2021 Jun 17;14:658339. doi: 10.3389/fnmol.2021.658339 (PMC8248683; doi:10.3389/fnmol.2021.658339)
Supplement: Supplementary file 7 [file Data_Sheet_1.PDF]

Supplementary Figure 1

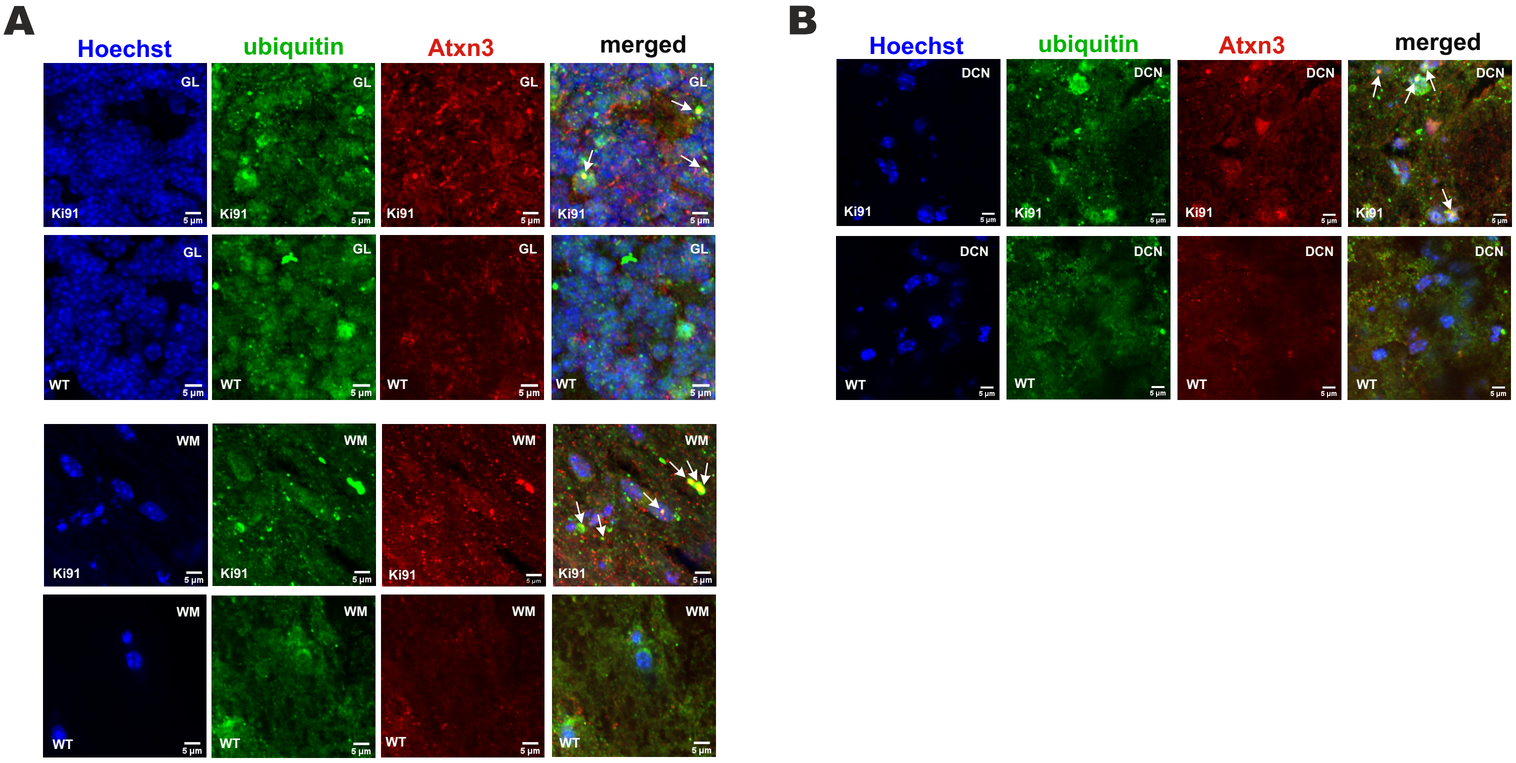

S1. The presence of ubiquintinated Atxn3 inclusions in the cerebellum of Ki91 SCA3/MJD

The ataxin-3 (red; 1H9) and ubiquitin (green; Z0458, Dako, Jena, Germany) co-immunostaining of the 18-month-old Ki91cerebellar sections revealed a number of inclusions positive for both Atxn3 and ubiquitin in the Ki91. Coherently, with Fig. 3, many aggregates positive for both Atxn3 (red; mouse anti-ataxin-3 antibody) and ubiquitin (green; rabbit anti-ubiquitin) were detected in granular layer (gl), white matter (wm) (A) and DCN (B) of the Ki91 cerebellum. Some of the inclusions are localized in the cell nucleus (blue; Hoechst 33342). White arrows indicate Atxn3- and ubiquitin-double positive inclusions. Scale bars: 5 µm. N=4 replicates; at least 4 pictures per brain region of each kind were collected.

Supplementary Figure 2

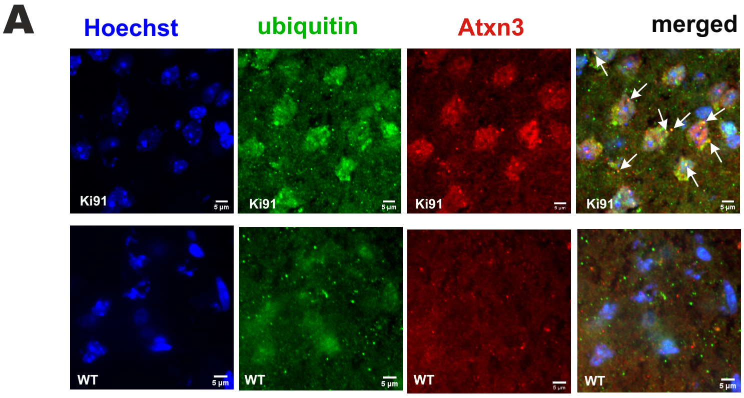

S2. The presence of ubiquintinated Atxn3 inclusions in the cerebral cortex of Ki91 SCA3/MJD

The ataxin-3 (red; 1H9) and ubiquitin (green; Z0458, Dako, Jena, Germany) co-immunostaining of the 18-month-old Ki91 brain sections revealed a number of inclusions positive for both Atxn3 and ubiquitin in the Ki91 cerebral cortex. Most of the aggregates positive for Atxn3 (red; mouse anti-ataxin-3 antibody) and ubiquitin(green; rabbit anti-ubiquitin) were detected in the cell nucleus (blue; Hoechst 33342). Scale bars: 5 µm. N=4 replicates; at least 4 pictures per brain region of each kind were collected.

Supplementary Figure 3

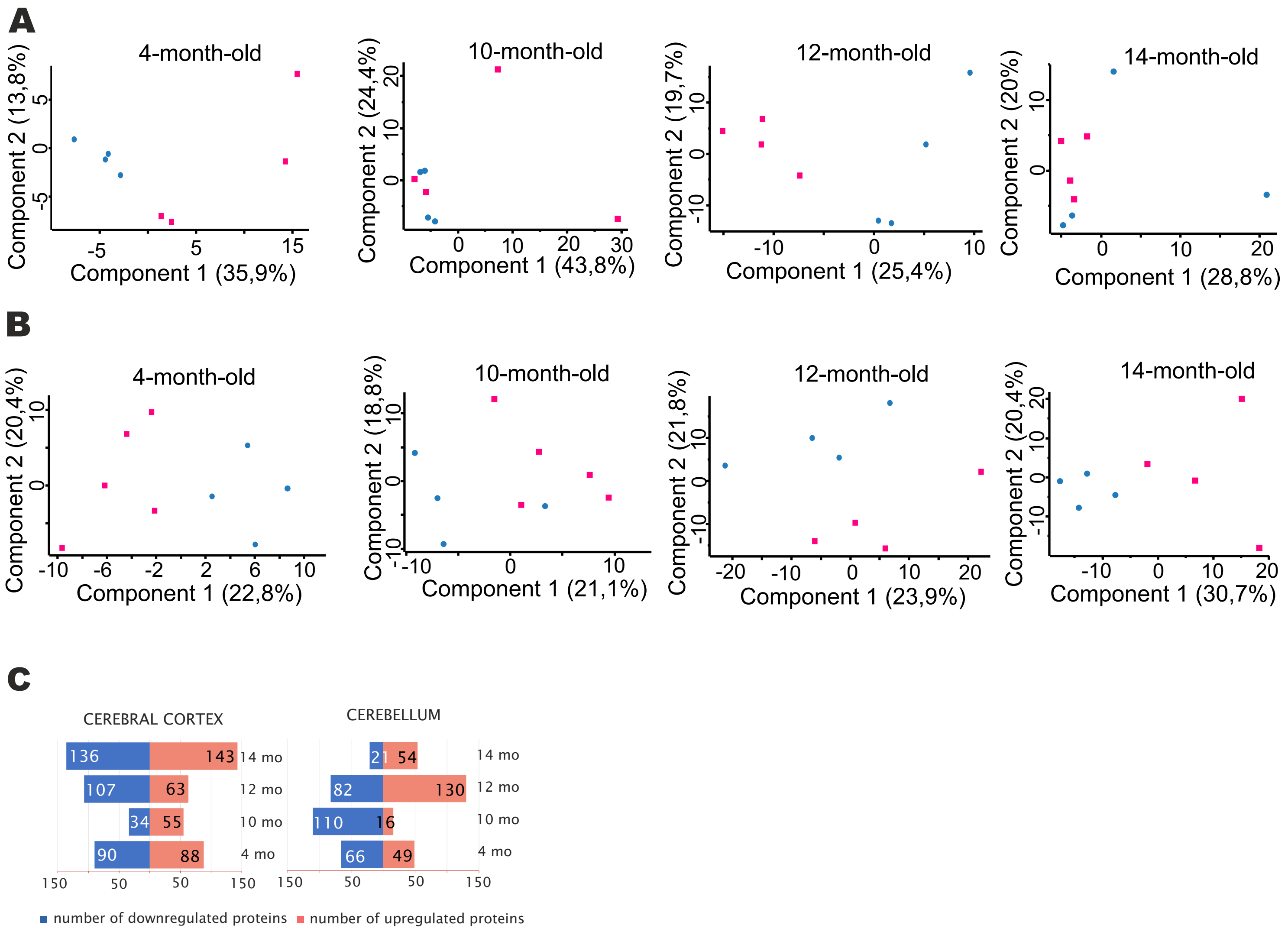

S3. Principal Component Analysis for proteomic data in the "parallel" approach of the cerebellum and cerebral cortex samples

Distinct clustering of dysregulated genes of proteomic data of the cerebellum (A) and cerebral cortex (B) of K91 and C57BL/6 mice (n=4-11 per genotype) is presented in the form of PCA graphs. The principal component analysis was performed in Perseus software ver. 1.6.1.3. Pink color denotes Ki91 brain samples, blue color C57BL/6 brain s amples. A total number of 1058 dysregulated proteins were identified in the cerebral cortex and 830 in the cerebellum ($p<0.05$; two-sample t-test) (C). There was no clear trend neither towards downregulation or upregulation.

Supplementary Figure 4

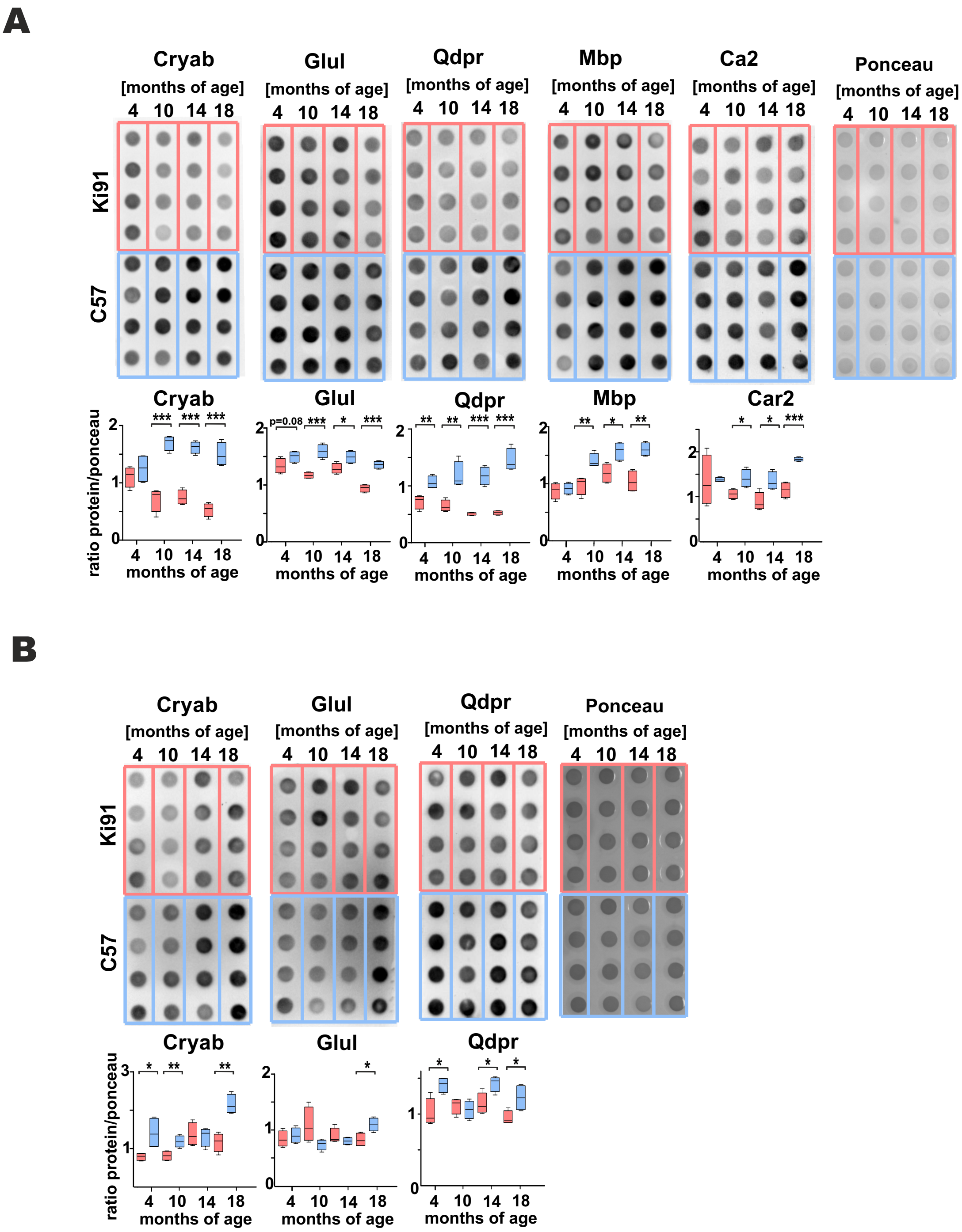

S4. Validation of dysregulated proteins identified in the LC-MS/MS proteomic "parallel" approach with vacuum dot blot assay in the Ki91 SCA3/MJD brain tissues

Vacuum dot blot analysis confirmed decreased levels of Cryab ($p < 0.001$; two-sample t-test), Glul ($p < 0.05$; two-sample t-test), Mbp ($p < 0.05$; two-sample t-test) and Car2 ($p < 0.05$; two-sample t-test) in the cerebral cortex of 10, 14, and 18-month-old Ki91 mice and Qdpr ($p < 0.01$; two-sample t-test) in the 4, 10, 14, and 18-month-old Ki91 mice (A). In the cerebellum of Ki91 animals, decreased levels of Cryab ($p < 0.05$; two-sample t-test) and Qdpr ($p < 0.01$; two-sample t-test) was shown in 4, 10, and 18-month-old Ki91 mice and decreased levels of Glul in 18-month-old mice (F). Ponceau was used as a loading control. N=4 per genotype, error bars: SEM. All experiments were performed in 3 technical replicates.

Supplementary Figure 5

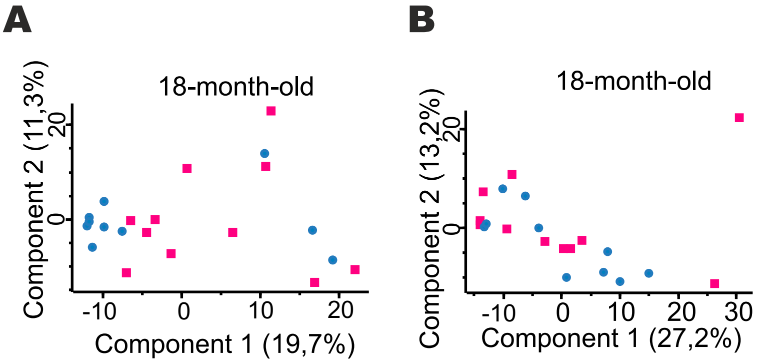

S5. Principal Component Analysis for proteomic data in the correlative approach of the cerebellum and cerebral cortex samples

A total number of 2555 proteins were identified in the cerebral cortex and cerebellum of 18-month-old mice (FDR <0.01). Distinct clustering of dysregulated genes of proteomic data of the cerebellum (A) and cerebral cortex (B) of K91 and C57BL/6 mice (n = 10 WT C57BL/6 and n=11 Ki91 mut/mut) is presented in the form of PCA graphs. The principal component analysis was performed in Perseus software ver. 1.6.1.3. Pink color denotes Ki91 brain samples, blue color C57BL/6 brain samples.

Supplementary Figure 6

A

Score	Symptoms
0	no motor dysfunction
1	few footslips during walking across rod
2	footslips with every (or almost every) step during walking
3	frequent footslips and loss of balance occuring during walking
4	frequent footslips and difficulties with movement, contractions of body
5	Impaired gait and severe loss of balance

B

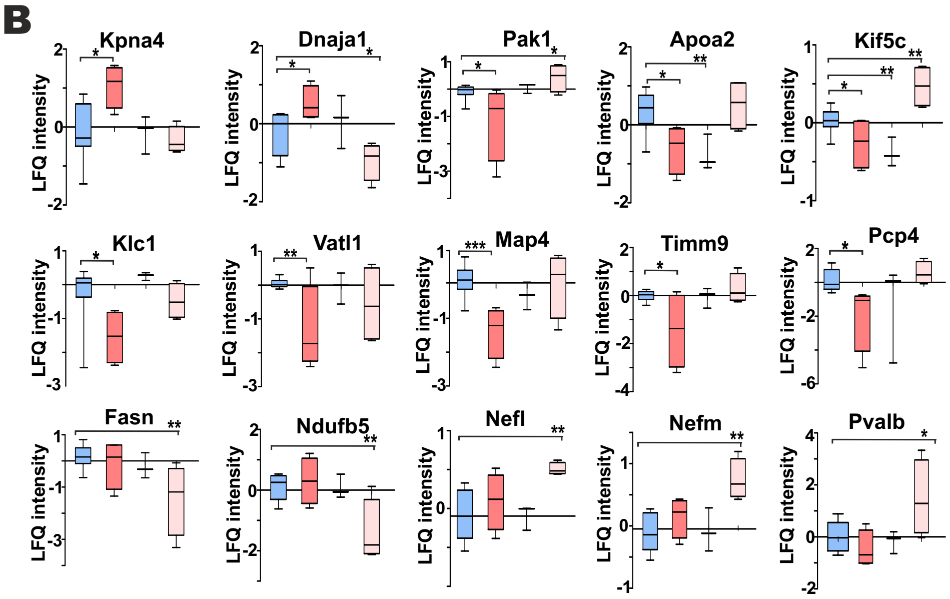

C

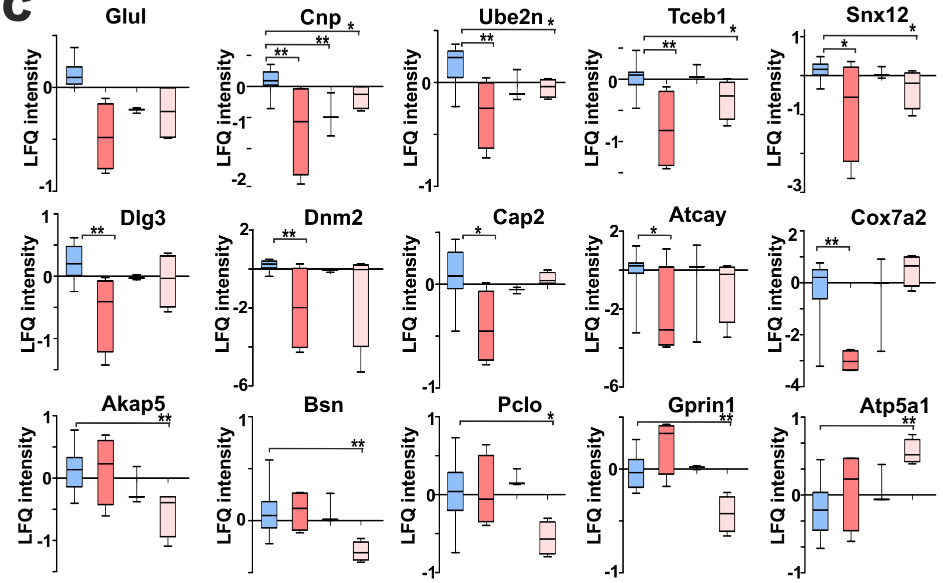

S6. Correlative proteomics on brains of Ki91 SCA3/MJD mice demonstrate dysregulated proteins involved in intracellular transport in severe SCA3 phenotype and mitochondria, cytoskeleton, and synaptic vesicles in mild phenotype.

We selected 3 phenotype subgroups in the Ki91 cohort during behavioral testing of 18-month-old mice: severe, moderate, and mild (n =4), based on score assessment evaluating motor impairments in the 0-5 scale (Kruskal-Wallis test, $p < 0.05$) (A). In the cerebellum derived from animals with a severe phenotype, there were upregulated ($p < 0.05$; two-sample t-test) heat shock protein Dnaja1 and a nuclear importin Kpna4 and downregulated proteins related to microtubule-based transport (Klc1, Kif5c, Vatl1, and Map4) and Purkinje cell protein (Pcp4) (B). In the cerebellum of animals with mild phenotype, neurofilament proteins were upregulated (Nefl, Nefm) together with Pvalb, Pak1, Kif5c ($p < 0.05$; two-sample t-test) (B). Mitochondrial proteins were downregulated in both severe (Timm9) and mild phenotypes (Ndufb5 and Fasn) (B). In the cerebral cortex of animals with severe phenotype proteins related to intracellular transport and cytoskeleton (Snx12, Dlg3, Dnm2, Cap2, and Atcay) and mitochondrial protein Cox7a2 were downregulated ($p < 0.05$; two-sample t-test) (C). Animals with mild phenotype displayed lower levels of proteins associated with synaptic vesicles and post-synaptic density (Akap5, Bsn, Pclo, Gprin1) (C). The blue color is for WT, and the gradient of pink color is for Ki91, the most intense pink color is a severe phenotype, and the palest pink color is for the mild phenotype. two-sample t-test (*$P < 0.05$, **$P < 0.01$, ***$P < 0.001$), n = 36, n = 4 per phenotype, error bars: SEM.

Supplementary Figure 7

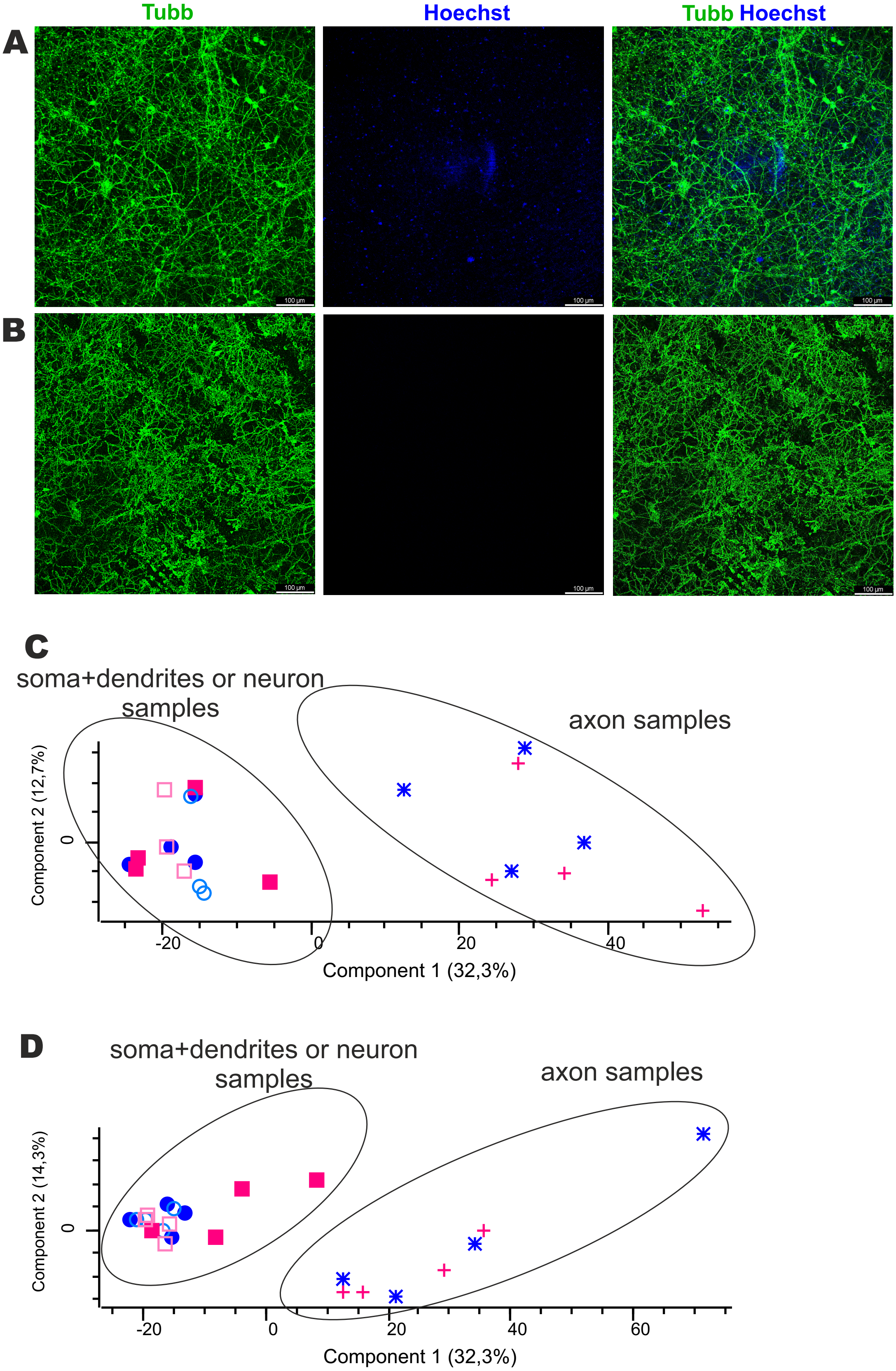

S7. Quality control of proteomic analysis of axonal transport

Clear separation of an axonal fraction from the somatodendritic fraction is demonstrated by immunostaining of culture filters isolated from the Boyden chamber before (A) and after scraping off the cell bodies (B), with the antibody for B-Tubulin (green) and nuclear dye Hoechst (blue). In addition, PCA graphs also show a clear separation between axonal and somatodendritic samples from cerebellar neurons (C) and cortical neurons (D).

Supplementary Figure 8

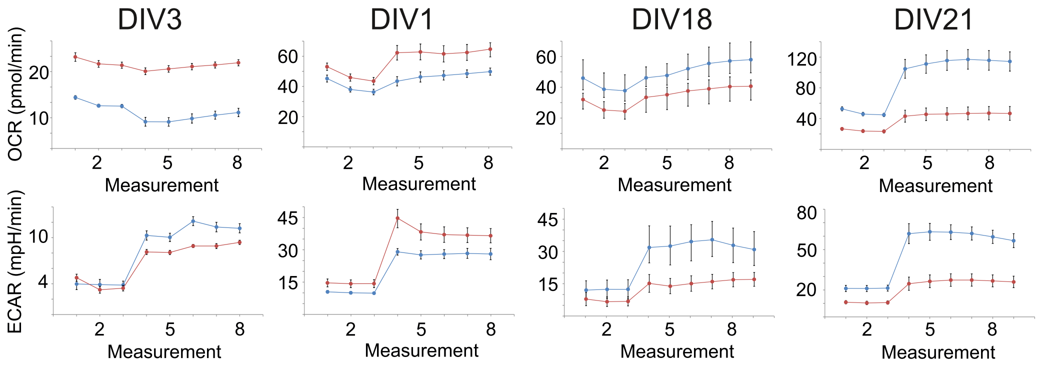

S8. Seahorse XFp Cell Energy Phenotype profiling in cerebellar neurons
The rate of mitochondrial respiration (OCR, oxygen consumption rate) and glycolysis (ECAR, extracellular acidification rate) were measured under baseline (3 measurements) and stressed conditions (5 measurements), which were caused by the injection of 1 μM of oligomycin and 1 μM of FCCP). The profiles of OCR (upper panel) and ECAR (lower panel) during testing are presented on the graphs.
